# Isolating the Role of Corticosterone in the Hypothalamic-Pituitary-Gonadal Transcriptomic Stress Response

**DOI:** 10.3389/fendo.2021.632060

**Published:** 2021-06-02

**Authors:** Suzanne H. Austin, Rayna M. Harris, April M. Booth, Andrew S. Lang, Victoria S. Farrar, Jesse S. Krause, Tyler A. Hallman, Matthew MacManes, Rebecca M. Calisi

**Affiliations:** ^1^ Department of Neurobiology, Physiology, and Behavior, University of California Davis, Davis, CA, United States; ^2^ Department of Molecular, Cellular and Biomedical Sciences, The University of New Hampshire, Durham, NH, United States; ^3^ Department of Biology, University of Nevada, Reno, Reno, NV, United States; ^4^ Department of Fisheries and Wildlife, Oregon State University, Corvallis, OR, United States

**Keywords:** corticosterone, acute stress, hypothalamic-pituitary-gonad axis, transcriptomics, birds (Aves)

## Abstract

Investigation of the negative impacts of stress on reproduction has largely centered around the effects of the adrenal steroid hormone, corticosterone (CORT), and its influence on a system of tissues vital for reproduction—the hypothalamus of the brain, the pituitary gland, and the gonads (the HPG axis). Research on the action of CORT on the HPG axis has predominated the stress and reproductive biology literature, potentially overshadowing other influential mediators. To gain a more complete understanding of how elevated CORT affects transcriptomic activity of the HPG axis, we experimentally examined its role in male and female rock doves (*Columba livia*). We exogenously administrated CORT to mimic circulating levels during the stress response, specifically 30 min of restraint stress, an experimental paradigm known to increase circulating CORT in vertebrates. We examined all changes in transcription within each level of the HPG axis as compared to both restraint-stressed birds and vehicle-injected controls. We also investigated the differential transcriptomic response to CORT and restraint-stress in each sex. We report causal and sex-specific effects of CORT on the HPG transcriptomic stress response. Restraint stress caused 1567 genes to uniquely differentially express while elevated circulating CORT was responsible for the differential expression of 304 genes. Only 108 genes in females and 8 in males differentially expressed in subjects that underwent restraint stress and those who were given exogenous CORT. In response to elevated CORT and restraint-stress, both sexes shared the differential expression of 5 genes, *KCNJ5*, *CISH*, *PTGER3*, *CEBPD*, and *ZBTB16*, all located in the pituitary. The known functions of these genes suggest potential influence of elevated CORT on immune function and prolactin synthesis. Gene expression unique to each sex indicated that elevated CORT affected more gene transcription in females than males (78 genes versus 3 genes, respectively). To our knowledge, this is the first study to isolate the role of CORT in HPG genomic transcription during a stress response. We present an extensive and openly accessible view of the role corticosterone in the HPG transcriptomic stress response. Because the HPG system is well conserved across vertebrates, these data have the potential to inspire new therapeutic strategies for reproductive dysregulation in multiple vertebrate systems, including our own.

## Introduction

Unpredictable perturbations or perceived threats in the environment can activate various endocrine cascades to promote behavioral and physiological coping mechanisms associated with survival. The hypothalamic-pituitary-adrenal (HPA) axis plays a central role in mediating a portion of these coping mechanisms (reviewed in 1). Parvocellular neurons in the hypothalamus of the brain release hormones such as arginine vasotocin (AVT; in birds and lizards) and corticotropin-releasing hormone (CRH) into the portal vasculature that access the anterior pituitary gland. There, AVT and CRH activate corticotrope cells to induce the release of adrenocorticotropic hormone (ACTH). ACTH travels through the bloodstream to the adrenal glands and binds to melanocortin 2 receptors (MC2R), which stimulates the synthesis of glucocorticoids [including corticosterone (*hereafter*, CORT) in adult birds and cortisol in many mammals]. CORT travels *via* the bloodstream to bind to its receptors, either nuclear or membrane bound, including the low affinity glucocorticoid receptor (GR) and high affinity mineralocorticoid (MR) receptor. The binding of CORT to nuclear receptors affects gene transcription throughout the body due to their global distribution (1). Receptor activation modifies metabolism, immune function, and induces behavioral and physiological changes (2–4).

When individual survival is favored over other energetically costly activities (3), the chronic depletion of bodily resources can inhibit other important biological processes (e.g., reproductive function, 2). The activation and regulation of reproduction and associated behaviors is mediated by a biological system of tissues consisting of the hypothalamus, pituitary, and gonads, referred to as the “HPG” axis. Its most well-studied endocrine cascade involves production and secretion of gonadotropin-releasing hormone (GnRH) from the preoptic area of the hypothalamus, which causes pituitary secretion of gonadotropins, luteinizing hormone (LH) and follicle stimulating hormone (FSH) (5). LH and FSH travel through the bloodstream and act upon receptors in the gonads, stimulating gametogenesis and the synthesis of sex steroids, such as testosterone and estradiol. These sex steroids then bind with receptors within the HPG axis to facilitate reproduction and sexual behaviors, and they also act as a negative feedback control mechanism.

It has long been known that exposure to certain stressors, and the subsequent physiological response, can negatively impact sexual behavior and reproduction. The activation of the HPA axis affects HPG function at multiple levels because MR and GR are globally distributed and are expressed throughout the body (6, 7). Elevation of CORT, a significantly influential and well-studied hormone in the stress response, can suppress the release of the reproductive hormone, GnRH, at the level of the brain, by interacting with gonadotropin inhibitory hormone [GnIH; also referred to as RFamide-related peptide (*RFRP)*], or with kisspeptin neurons (in mammals) (8). This, in turn, reduces the rate of depolarization of the GnRH-1 neuron and subsequent GnRH-1 release, which causes the reduction of LH, FSH, and steroidogenic capacity and gametogenesis. Thus, activation of the HPA axis in response to environmental perturbations has the ability to affect each endocrine-producing tissue of the HPG axis, and therefore, reproduction.

Research on the influential role of CORT on the HPG axis has predominated much of the stress and reproductive biology literature, which has potentially overshadowed other influential mediators of stress. In this study, we used the model of the rock dove (*Columba livia*) to experimentally test the extent to which changes in gene expression within the HPG axis were explained by an increase in circulating CORT – a key characteristic of a stress response. Previously, our group described the genomic transcriptome community of sexually mature, non-breeding male and female doves (9). Then, we tested how gene transcription within the HPG axis of both sexes was affected by activation of the HPA axis, the latter which we stimulated using a common restraint stress paradigm in which subject mobility is restricted for 30 min (10). We reported a heightened and mostly sexually specific genomic stress response throughout the HPG axis. In this study, we isolated the role of elevated CORT in both sexes using the same restraint-stress paradigm to determine its causal and sex-typical effects on HPG gene transcription. We did this by exogenously administrating CORT to mimic circulating levels following 30 min of restraint stress. We then compared differential gene expression within the HPG following 30 min of exogenous CORT and 30 min of restraint stress. We report stress-induced and sex-specific changes in HPG transcriptomic activity caused by elevated circulating CORT. We predicted a reduction in differentially expressed genes in response to exogenous CORT compared to restraint stress.

## Materials and Methods

Subject housing, sampling and analysis procedures used in this study replicated those used in Calisi et al. (10) unless otherwise noted. These methods are described in brief in the following text.

### Housing

Animals were socially housed at the University of California, Davis, in large semi-enclosed outdoor aviaries (1.5 x1.2 x2.1 meters). Each aviary had 16 nest boxes and approximately 8 sexually reproductive adult pairs and their dependent offspring. Food (Farmer’s Best Turkey/Game Bird Starter Crumbles (27% crude protein, 1.2% lysine, 0.3% methionine, 4% crude fat, 5% crude fiber, 0.8% phosphorous, 1-1.5% calcium, 0.3-0.6% NaCl, 0.25% Na; Farmers Warehouse Company, Keves, CA, USA), Farmer’s Best Re-cleaned Whole Corn (7.5% crude protein, 3.4% crude fat, 3.5% crude fiber, 1.7% ash; Farmers Warehouse Company, Keves, CA, USA), and red grit (33-35% calcium, 0.01% phosphorous, 0.05-0.08% Salt; Volkman Seed Factory, Ceres, CA, USA), water, and nesting material was provided *ad libitum*. Birds were exposed to natural light, which was augmented with artificial fluorescent lights set to a 14L:10D cycle. All birds collected in this study were between 5 months and 2 years old, sexually mature, and were not actively breeding at the time of collection.

### Corticosterone Solution

CORT (0.2mg/ml, Sigma 4-Pregnene-11beta-diol-3,20-dione, C-2505, Lot 092K1255) was dissolved by vortexing it in a peanut oil vehicle (11) one day prior to its administration and stored in a 15 mL centrifuge tube at room temperature. The concentration used to simulate circulating levels of endogenous CORT in response to restraint stress was informed by preliminary validation trials.

### Animal and Tissue Collections Methods

Collections occurred between 0900-1200 (PST) following animal care and handling protocols (UC Davis IACUC permit # 20618). Subjects were sexually mature and did not have an active nest. They were randomly assigned to either the vehicle-control group (hereafter, *control*, 8 females, 5 males received the peanut oil vehicle only) or CORT treatment group (8 females, 8 males received CORT mixed with peanut oil). To administer treatments, subjects were captured in ≤1 min of entering their aviary, transported by hand to an adjacent room, and immediately injected with either a control or CORT solutions intramuscularly (pectoralis muscle). Subjects were returned to their aviaries <5 min post initial disturbance and left undisturbed for 30 min, after which they were re-captured and immediately anesthetized using isoflurane (<2 min) prior to decapitation. Trunk blood was immediately collected after decapitation, brains were flash frozen on dry ice, and pituitaries and gonads were submerged in RNALater (Invitrogen, Thermo Fisher Scientific, REF: AM7021) at collection before freezing them on dry ice. All tissues were then transferred to a -80°C freezer and stored until analyses.

In the laboratory of Dr. Rebecca Calisi at the University of California, Davis, brains were sectioned coronally on a Leica CM 1860 cryostat at 100 µm to allow for precise biopsy of hypothalami and lateral septa, replicating our previous collections (9, 10). Biopsied hypothalamic tissue was submerged in RNALater and shipped overnight on dry ice to the lab of Dr. Matthew MacManes at the University of New Hampshire for further processing. Trunk blood was centrifuged at 4°C for 10 minutes and plasma was aspirated and stored at -80°C.

### Hormone Assays

Plasma was assayed for circulating CORT concentrations using radioimmunoassay (RIA; see 10) using a dilution of 1:20 in a commercially available CORT RIA kit (MP Biomedicals, Orangeburg, NY) to confirm an increase in circulating CORT levels (ng/mL) in response to exogenous CORT and restraint stress treatments. The assay was validated for cross-reactivity to assess the potential for interference from other plasma compounds, and the limit of detection was estimated at 0.0385 ng/mL.

Two-way ANOVAs were conducted to assess differential circulating concentrations between groups (α = 0.05; lme4 v1.1-17, R v3.5.1), and *post-hoc* pairwise comparisons were conducted with a Dunnett adjustment for multiple comparisons (emmeans v.1.2.3). Prior to analysis, CORT values were ln-transformed to normalize the distribution of the data in order to meet model assumptions. Back-transformed estimates (e^estimate^) are presented in the results, which should be interpreted as a magnitude difference between groups.

### Illumina Library Preparation and Sequencing

Tissues frozen in RNALater were thawed on ice in an RNAse-free work environment. Total RNA was extracted using a standard Trizol extraction protocol (Thermo Fisher Scientific, Waltham, MA), and RNA quality was assessed using the Tapestation 2200 Instrument (Agilent, Santa Clara, CA). Illumina sequence libraries were prepared using the TruSeq RNA Stranded LT Kit (Illumina, San Diego, CA), and library quality assessed using the Tapestation 2200 Instrument (Agilent, Santa Clara, CA). Each library was diluted to 2nM with sterile purified commercially available molecular biology-grade water (VWR) and pooled in a multiplexed library sample. The multiplexed library sample was then sent to the Novogene company for 125 base pair paired-end sequencing on a HiSeq 4000 platform.

### Transcriptome Assembly Evaluation and Improvement

The previously constructed Rock Dove transcriptome version 1.0.4 assembly (10) was evaluated to ensure that transcripts expressed uniquely in the stress condition were included. To accomplish this, reads from the pituitary, hypothalamus, and gonads from one stressed male and one stressed female were assembled following the Oyster River Protocol (12). Unique transcripts contained in this assembly relative to the previously described assembly were identified *via* a BLAST procedure. Novel transcripts, presumably expressed uniquely in the stress condition were added to this existing assembly, thereby creating the Rock Dove v. 1.1.1 transcriptome (available at https://s3.amazonaws.com/reference_assemblies/Rockdove/transcriptome/RockDove.HPG.v1.1.1.fasta). This new assembly was evaluated for genic content *via* comparison with the BUSCO version 2.0 Aves database (13).

### Mapping and Global Analysis of Differential Gene Expression

Reads were quality and adapter trimmed to a Phred score =2 using the software package Trimmomatic (14). Reads were then quasimapped to the Rock Dove transcriptome (v. 1.1.1) after an index was prepared using Salmon 0.9.0 (15). Rock dove transcript IDs were mapped to genes from the *Gallus gallus* genome (v. 5), using BLAST (16). All data were then imported into the R statistical package (v. 3.3.0) (17) using tximport (18) for gene level evaluation of gene expression, which was calculated using contrasts that separately compared treatment group (control vs. CORT treatment) for each tissue (hypothalamus, pituitary, gonads) and sex (male or female) using edgeR (v. 3.5.0) (19) following TMM normalization and correction for multiple hypothesis tests by setting the false discovery rate (FDR) to 1%. Differential expression values resulting from 30 min of restraint stress were taken directly from Calisi et al. (10). Briefly, contrasts comparing treatment groups (baseline control vs. restraint stress treatment) were calculated as above for each tissue and sex (see 10 for more details).

### Candidate Gene Expression Evaluation

A set of 47 genes that target specific research questions was selected *a priori* for evaluation based on their known involvement in reproduction and in association with the stress response or CORT feedback (**Table 1**). Normalized values of gene expression were compared by treatment group. Differences in gene expression of candidate genes in the hypothalamic, pituitary, and gonadal tissues were compared in response to CORT treatment as compared to controls (expression ~ treatment) or involved a re-analysis of restraint-stress and baseline controls from Calisi et al. (10) using a robust regression model framework (alpha=0.05; rlm, Package MASS v7.3-50; robtest, Package sfsmisc v1.1-2; R v3.5.2 (20). These analyses were conducted separately for each sex.

**Table 1:** *A priori*-identified stress and reproduction-associated target genes that differentially express in response to CORT and restraint stress treatments (α= 0.05).

Gene name	Gene abbreviation	Hypothalamus		Pituitary		Testes	Ovary
		M	F	M	F	M	F
**STRESS AXIS**							
Arginine vasotocin	*AVP*					▽	
Arginine vasotocin-like receptor 1A	*AVPR1A*	▲					
Arginine vasotocin-like receptor 1B	*AVPR1B*						
Vasotocin-like receptor	*AVPR2*						▲
Corticosteroid Binding Globulin	*SERPINA4*						
Corticotropin Releasing Hormone	*CRH*						▲
Corticotropin Releasing Hormone Receptor 1	*CRHR1*					▲	▽
Dopamine receptor D1	*DRD1*						
Dopamine receptor D2	*DRD2*				▲		
Dopamine receptor D3	*DRD3*						
Dopamine receptor D4	*DRD4*						▽
Dopamine receptor D5	*DRD5*						
GABRQ gamma-aminobutyric acid (GABA) A receptor, theta	*GABRQ*		▲				
Glucocorticoid receptor	*NR3C1*						▲
11-beta hydroxysteroid dehydrogenase 1a	*HSD11B1a*		▲				
11-beta hydroxysteroid dehydrogenase 1B	*HSD11B1b*						
11-beta hydroxysteroid dehydrogenase 2	*HSD11B2L*			▲		▲	
11-beta hydroxysteroid dehydrogenase 1 like	*HSD11B1L*						
Melanocortin 2 receptor	*MC2R*				▽	▽	
Mineralocorticoid receptor	*NR3C2*						
Oxytocin-like receptor	*OXTR*					▽	
Proopiomelanocortin	*POMC*						
**REPRODUCTIVE AXIS**							
Androgen receptor	*AR*	▲		▽			
Aromatase CYP19A1	*CYP19A1*			▽			
ER alpha	*ESR1*						
ER beta	*ESR2*						
FSH Receptor	*FSHR*			▲			
Follicle stimulating hormone beta subunit	*FSHB*	▽					
Ghrelin/obestatin prepropeptide	*GHRL*						
Galanin	*GAL*						
Gonadotropin inhibitory hormone	*NPVF*	▽				▽	
Gonadotropin inhibitory hormone receptor	*NPFFR1*	▽					
Gonadotropin releasing hormone I	*GNRH1*				▲		
Gonadotropin releasing hormone I receptor	*GNRHR*						
Leptin receptor	*LEPR*						
Luteinizing hormone/ choriogonadotropin receptor	*LHCGR*						▲
Progesterone receptor	*PGR*		▽				
Prolactin	*PRL*						
Prolactin receptor	*PRLR*	▲					
Vasoactive Intestinal Peptide	*VIP*		▲				▽
Vasoactive Intestinal Peptide Receptor 1	*VIPR1*						
**THYROID AXIS**							
Deiodinase iodothyronine type I	*DIO1*						
Deiodinase iodothyronine type II	*DIO2*		▽				
Deiodinase iodothyronine type III	*DIO3*						
Thyroid hormone receptor alpha	*THRA*						
Thyroid hormone receptor beta	*THRB*					▽	
Thyroid stimulating hormone receptor	*TSHR*						
KEY							
						CORT upregulated	
						CORT downregulated	
▲						Restraint stress upregulated	
▽						Restraint stress downregulated	
▽						Dashed line: CORT & restraint stress	

Pink denotes upregulated activity in CORT-treated birds and dark blue denotes downregulated activity in CORT-treated birds. Solid upward facing triangles denote upregulated in restraint stress birds, and open downward facing triangles denote downregulated in restraint stress birds. Dashed lines denote a significant change in gene expression in both restraint stress and CORT-treated birds.

In Calisi et al. (10), a similar candidate gene analysis was performed using generalized linear models; however, due to the high variance of the *y*-variable (i.e., normalized gene expression) for a number of candidate genes, and the overall smaller sample size in the current study, we deemed robust regression a more appropriate analytical approach. Briefly, robust regression is a type of analysis that both incorporates and reduces the influence of outliers on results without discounting their contributions. This is a more conservative approach, and thus, can increase the chance of false negatives, particularly when effect sizes are small. To make the previous work on restraint-stress directly comparable with the results from current analyses, and to remain internally consistent within the current study, we chose to re-analyze gene expression of the target genes from the restraint-stress dataset using the methodology we deemed most appropriate for the current study. We also expanded on the original candidate gene list from Calisi et al. (10) to include a number of genes associated with CORT activation or de-activation, which were of particular interest in the current study. Because we used a different, more conservative analytical approach to analyze the CORT dataset that then required us to re-analyze the restraint stress dataset, there were some differences in statistical significance in the current study than were originally reported in Calisi et al. (10). Any data we report that differ between those reported in Calisi et al. (10) and this re-analysis can be attributed to the use of a different analytical approach.

### Isolating the Role of CORT in the HPG Transcriptomic Stress Response

We compared genes that differentially expressed in response to CORT treatment (as compared to vehicle-injected controls) to those that differentially expressed in response to restraint-stress treatment [as compared to non-stressed controls; the latter results we reported in Calisi et al. (10)]. Genes identified as differentially expressed in response to both experimental manipulations (CORT and restraint-stress treatments) as compared to their respective controls (vehicle control and unstressed treatments) were considered the genes within the HPG axis whose response to the stress stimuli was most likely due to elevated circulating concentrations of CORT.

## Results

### Corticosterone

**Figure 1:**
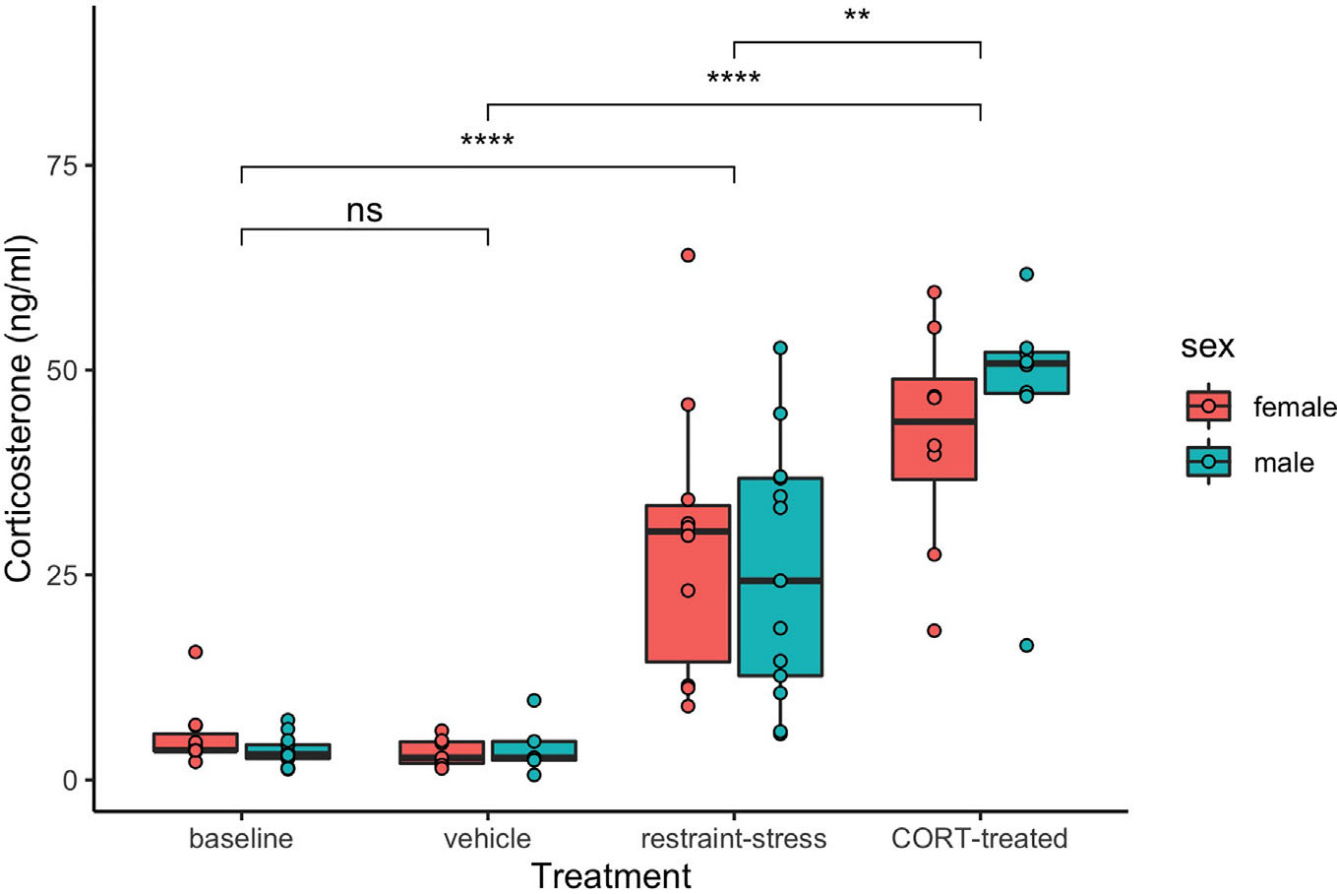
Circulating corticosterone (ng/mL) of male (aqua) and female (pink) *C. livia* of restraint-stress (baseline control and restraint-stress, left panel) compared to the exogenous treatment (vehicle and CORT-treated, right panel). Boxes include medians and interquartile range while whiskers indicate variability outside the quartile range. Statistically significant differences between treatments are indicated by brackets and ‘ns’ = *p* > 0.05, ‘**’ = *p* < 0.01, ‘****’ = *p* < 0.001).

Both restraint stress and exogenous CORT treatment increased circulating levels of CORT in both sexes (treatment: *F*_3, 71_ = 83.4, *P* < 0.001, sex: *F*_1, 71_ = 0.4, *P* = 0.279; **Figure 1**). Circulating CORT concentrations did not differ between the control group of our previous restraint stress study and our current study [restraint-stress control group vs. CORT control group: least-squares mean back-transformed estimate (± SE) = 1.31 ± 0.22, (Dunnett-adjusted) *P* = 0.631; CORT treatment vs. vehicle control=14.69 ± 0.23, *P* < 0.001; restraint-stress treatment vs. CORT treatment= 0.53 ± 0.20, *P* < 0.001]. Median circulating CORT concentrations were higher in the exogenous CORT treatment group as compared to our previous restraint-stress treatment group (47.05 ng/mL ± 13.34 SD vs. 29.8 ng/mL ± 16.26 SD), though there was substantial overlap of values (restraint stress range: 1.3 – 64 ng/mL; **Figure 1**).

### Sequence Read Data and Code Availability

In total, hypothalami, pituitary, and gonads (testes or ovaries) from 11 males and 16 females were sequenced (n=81 samples in total). Each sample was sequenced with between 6.6 million and 23.5 million read pairs. Read data are available using the European Nucleotide Archive project ID PRJEB28645. Codes used for the analyses of these data are available at https://github.com/macmanes-lab/RockDove/tree/master/CortStudy.

### Transcriptome Assembly Characterization

The Rock Dove v. 1.1.1 transcriptome contains 97,938 transcripts, of which 5,133 were added as part of this study to the previous version 1.1.0 transcriptome. This newly compiled transcriptome data improves genic contiguity, increasing the number of complete BUSCOs 1.4% to achieve 87.5% relative to the v. 1.1.0 assembly.

### Sequence Read Mapping and Estimation of Gene Expression

Raw sequencing reads corresponding to individual samples of hypothalami, pituitary glands, and gonads were mapped to the *C. livia* reference HPG axis transcriptome (v. 1.1.1) using Salmon, resulting in 80% to 90% read mapping. These data were imported into R and summarized into gene-level counts using tximport, after which, edgeR was used to generate normalized estimates of gene expression. A total of 14,938 genes or their isoforms were expressed in HPG tissues.

### Evaluation of Transcriptomic Expression

**Figure 2:**
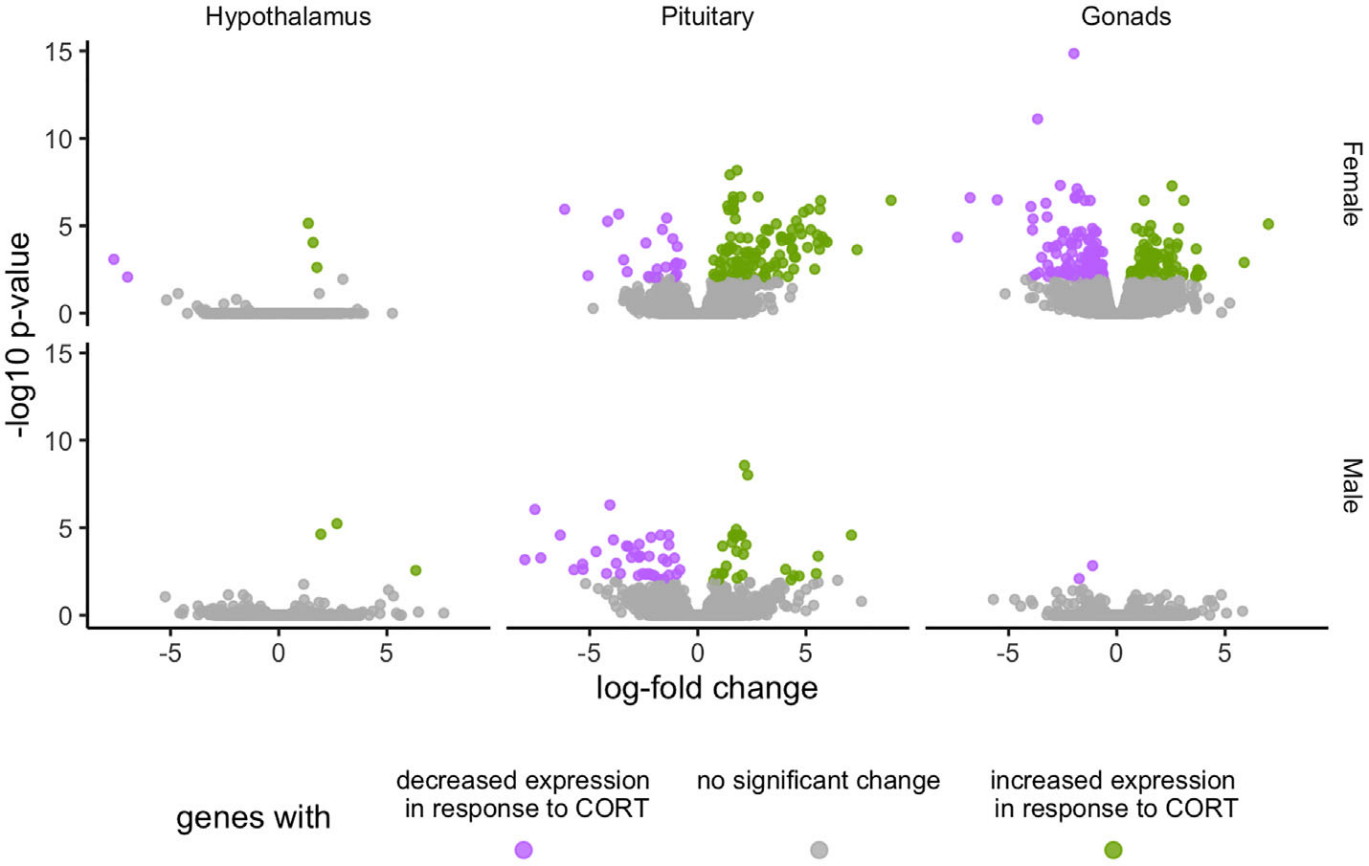
Volcano plots depicting total transcriptional changes of CORT-treatment group as compared to vehicle-control group. Volcano plots represent the number of differentially expressed genes throughout the HPG axis of females (top) and males (bottom). The x-axis represents log-fold change of differentially expressed genes and the y-axis -statistical significance (log10 p-values where FDR <0.01). Each datapoint represents a differentially expressed gene in the HPG axis. Significant differences in gene expression are represented in green (increased expression) or purple (decreased), or gray (no significant difference) in response to corticosterone treatment.

Global patterns of gene expression were analyzed using edgeR to assess the HPG transcriptomic response to experimentally elevated circulating CORT. After controlling for > 35,000 multiple comparisons, the count data were normalized using the TMM method (21, 22), which, in brief, uses a set of scaling factors for library sizes to minimize inter-sample log-fold changes for most genes. This analysis revealed a significant transcriptomic response of the HPG axis to CORT treatment, with females experiencing more differential expression as compared to males (**Figure 2**). All differentially expressed genes in female and male HPG tissue in response to our 30 min CORT treatment can be found at https://cortstudy.page.link/DEresults.

### Unique Differential Gene Expression in Response to Exogenous CORT Treatment

**Figure 3:**
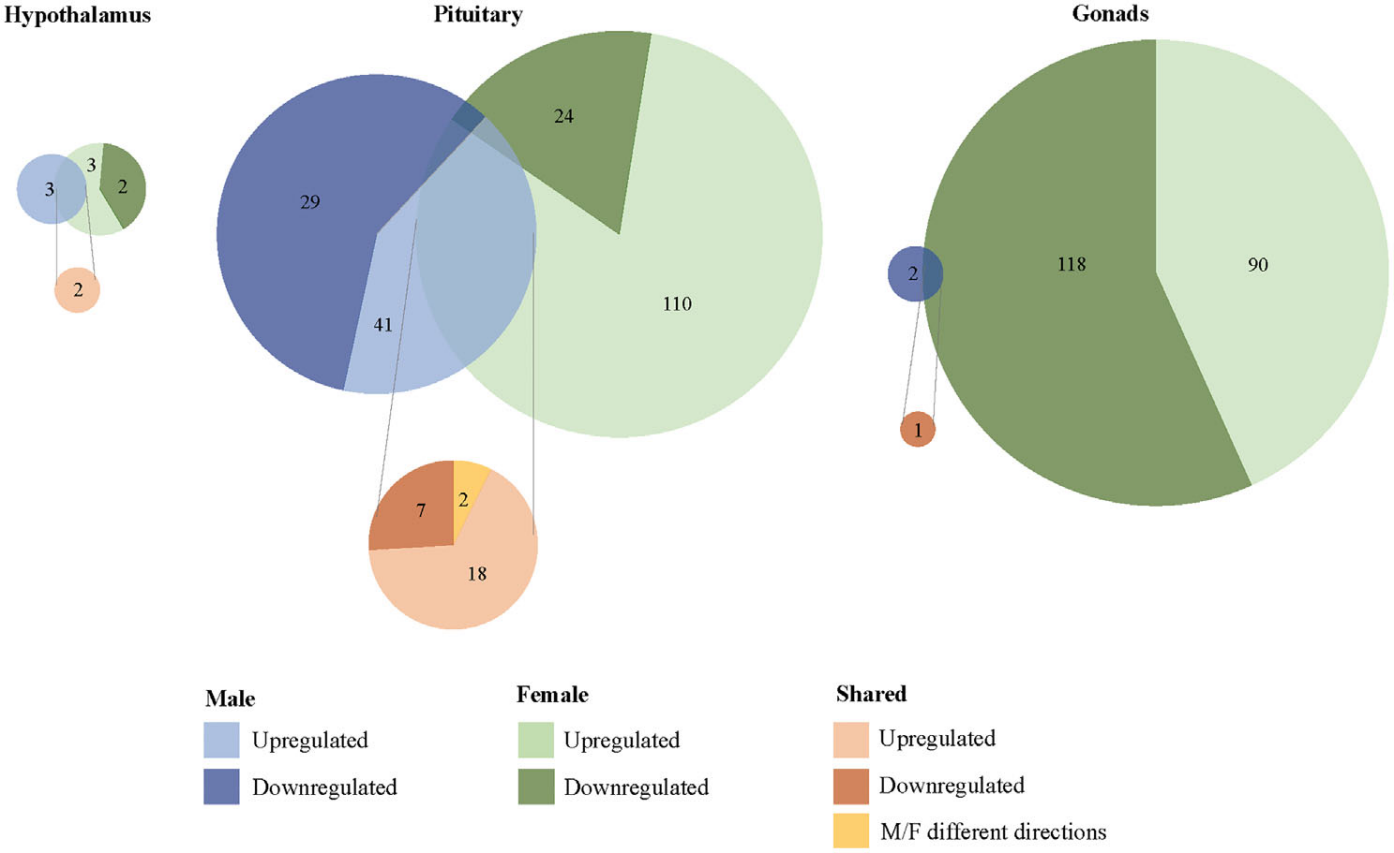
Sex-biased differential transcriptomic expression in response to exogenous CORT treatment. A weighted Venn diagram depicting the overlap of the number of differentially expressed genes between the sexes in the hypothalamus, pituitary, and gonads in response to CORT as compared to vehicle. Genes that upregulated in expression in response to CORT are depicted by a lighter shade; genes that down-regulated in response are depicted by a dark shade. Numbers within shaded areas indicate the number of CORT-responsive genes.

We found 304 genes responsive to our exogenous CORT treatment that were not responsive to our restraint stress treatment. Of these genes, 6 exhibited differential expression patterns in the hypothalamus (females: 5, males: 3), 179 in the pituitary (females 136, males 71), and 209 in the gonads (females 208, males 2) (**Figure 3**; **SI 2: Tables 1–6**). Across all tissues, the differential expression of 38 genes was shared between the sexes in response to exogenous CORT treatment (hypothalamus: 2, pituitary: 28, and gonads: 1; **SI 2: Table 7–9**).

### Unique Differential Gene Expression in Response to Restraint Stress

**Figure 4:**
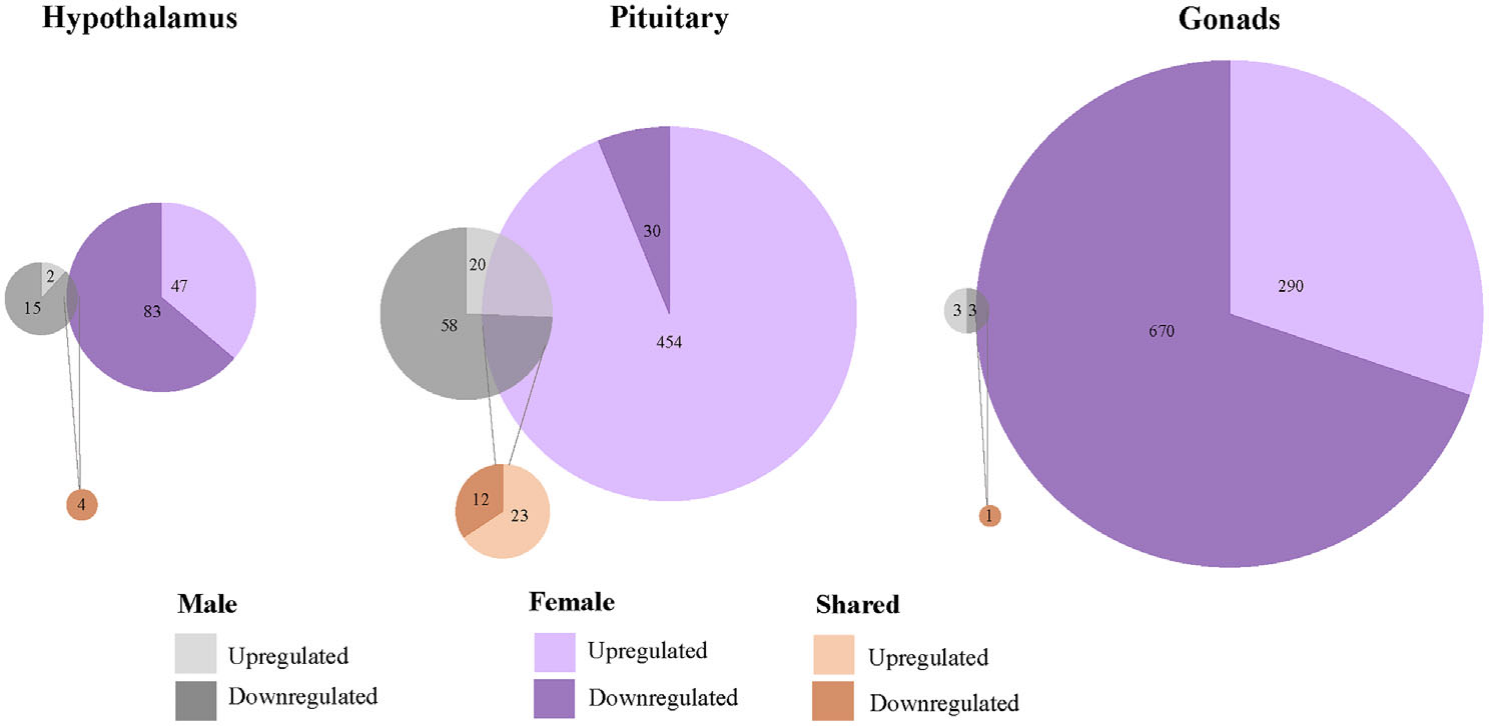
Sex-biased differential genomic expression that uniquely responded to restraint-stress treatment but not CORT treatment. A weighted Venn diagram depicting the overlap of the number of differentially expressed genes between the sexes in the hypothalamus, pituitary, and gonads that uniquely responded to restraint stress and not CORT treatment as compared to controls. Genes that upregulated in expression in response to CORT are depicted by a lighter shade; genes that down-regulated in response are depicted by a dark shade. Numbers within shaded areas indicate the number of restraint stress-responsive genes.

We found that 1567 genes differentially expressed uniquely in response to restraint stress as compared to those treated with exogenous CORT. Of these, 147 genes were differentially expressed in the hypothalamus (females: 130, males: 17), 562 in the pituitary (females 484, males 78), and 966 in the gonads (females 960, males 6) (**Figure 4**). Across all tissues, the differential expression of 38 genes was shared between the sexes in response to restraint stress treatment (hypothalamus: 1, pituitary: 35, gonads: 1). Restraint stress specific genes can be found in **Tables 1–9** of the **SI 3**.

### Isolation of Restraint Stress-Responsive Gene Activity Attributed to Circulating CORT Elevation

#### Changes in Expression Shared by Both Sexes

**Figure 5:**
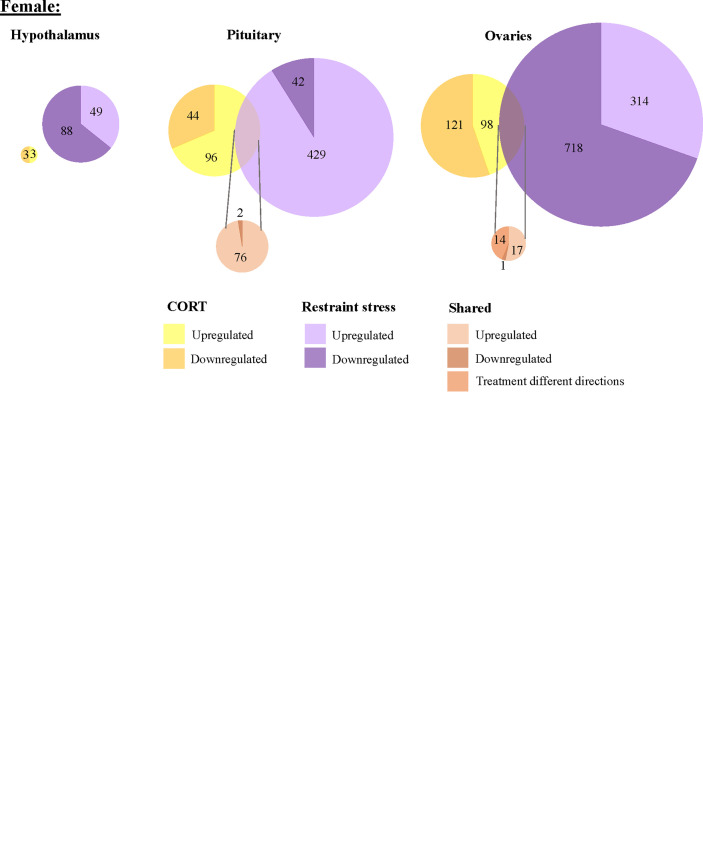
Shared and unique gene expression responses to CORT treatment and restraint-stress. Weighted Venn diagrams of significantly differentially expressed genes for male (top) and female (bottom) of CORT-treated and restraint stress treatments (data from Calisi et al. (10) in the hypothalamus, pituitary and gonads. Circles are proportional within males and females, but not between.

To isolate the effects of CORT on the HPG transcriptomic stress response observed after 30 min of restraint stress, we identified shared differential expression resulting from both exogenously administered CORT and restraint stress as compared to their respective controls. In both the male and female pituitary, 5 genes differentially expressed in exogenous CORT and restraint-stress treated birds: *KCNJ5*, *CISH*, *PTGER3*, *CEBPD*, and *ZBTB16*. We report the results from our differential analysis [log-fold change (logFC) and false discovery rate (FDR)] with a brief description of known gene functionality in vertebrates in **SI Table 1**, **Figure 5**. There were no shared genes between males and females at the level of the hypothalamus or gonads that responded to both exogenous CORT and restraint-stress treatments.

#### Changes in Expression Unique to Each Sex

Within the HPG axis, we identified 108 genes unique to females (including 5 isoforms) and 8 genes unique to males whose response to 30 min of restraint stress was attributed to circulating CORT elevation. In females, 78 of these genes in the pituitary (**SI 1: Table 3**) and 32 genes in the ovaries (**SI 1: Table 4**) were significantly differentially expressed in CORT and restraint-stress individuals. *CEPBD* and *TSC22D3* differentially expressed in both the pituitary and ovaries and are therefore included in both tissue gene counts. In males, 8 genes were isolated in pituitary. Only 3 of these genes uniquely differentially expressed in males, *KLF9*, *SLAINL*, and *PVALB* (**SI 1: Table 2**). We did not identify any restraint stress-induced gene activity explained by exogenous CORT elevation within the hypothalamus of either sex or in the testes. A brief summary of gene function for these differentially expressed genes is also provided in **SI 1: Tables 2–4**.

### Candidate Gene Expression Evaluation

**Figure 6:**
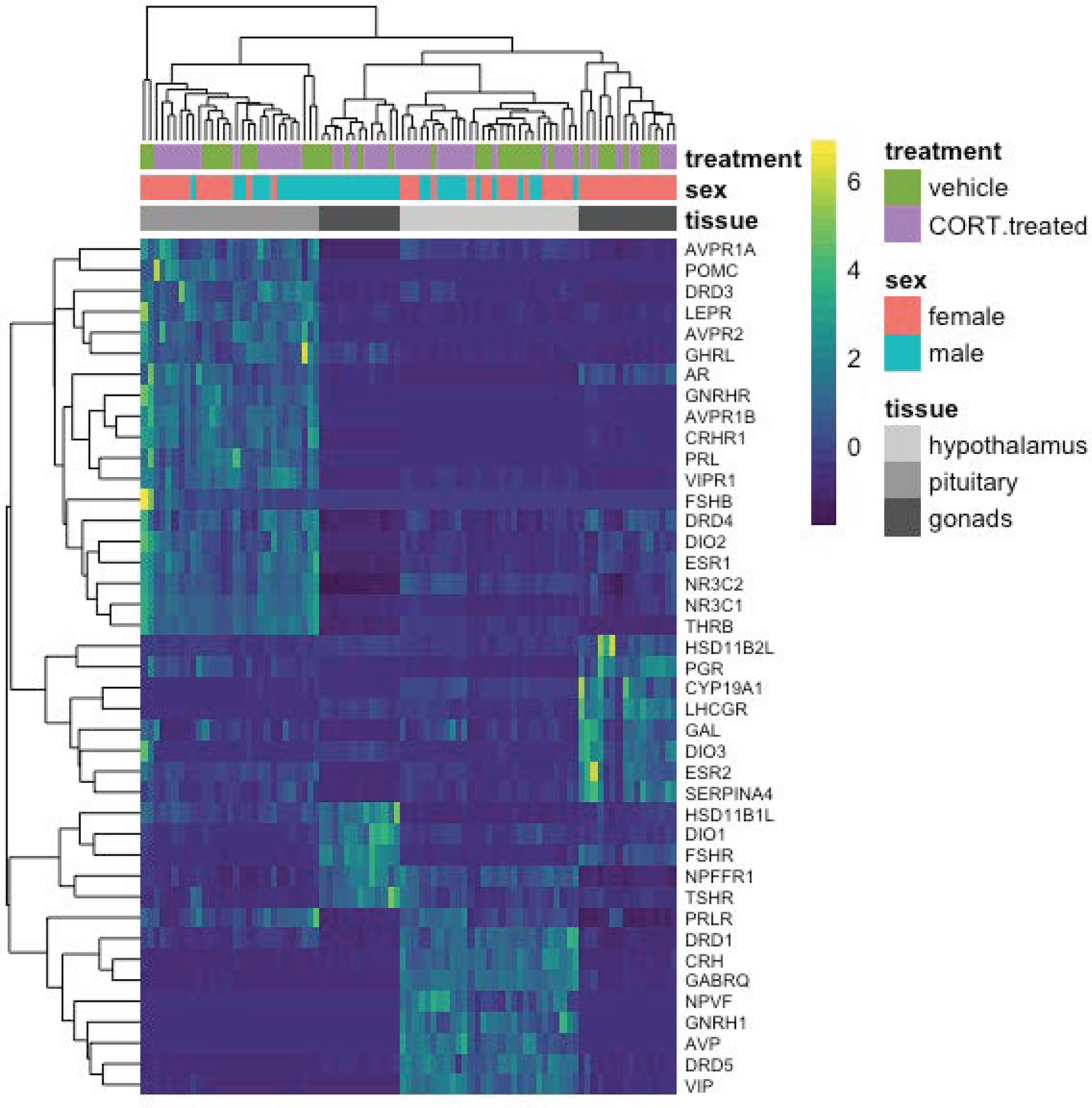
A heatmap depicting individual candidate gene expression values (normalize gene counts) represented as colors. Blue shades signify low levels of expression; Yellow shades signify relatively higher levels of expression. The *Y*-axis on the right denotes the gene abbreviation; the *Y*-axis on the left is a dendrogram of expression similarity. The *X*-axis denotes the sex, tissue, and treatment group of the animal (CORT-treated or the vehicle control).

To detect more subtle changes in gene expression, we conducted a separate analysis to include only a select group of *a priori*-targeted genes (*N* = 47) based on their previously known role in the stress response or reproductive function (**Figure 6**; **Table 1**). We identified two candidate genes, *DIO2* in the female hypothalamus and *PRLR* in the male hypothalamus, for which expression varied in response to 30 min of restraint stress could be explained by elevated CORT.

Most target genes responded uniquely to either exogenous CORT or restraint stress treatments. As compared to vehicle-injected controls, exogenous CORT treatment resulted in the differential response of 22 candidate genes: 12 in the hypothalamus (male: 10, female: 3), 12 in the pituitary (male: 8, female: 7), and 9 in the gonads (male: 7, female: 3). While restraint stress compared to its control resulted in the differential response of 26 candidate genes: 11 in the hypothalamus (male: 6, female: 5), 7 in the pituitary (male: 4, female: 3), and 12 in the gonads (male: 6, female: 7).

## Discussion

A substantial body of research has been dedicated to understanding the critical role CORT plays *via* the stress response in promoting survival (1–4) and its subsequent consequences in other biological processes, like reproductive function (2, 6, 7, 10). In this study, we experimentally tested the extent to which changes in gene activity in the HPG axis could be explained by an increase in circulating CORT that is characteristic of an acute stress response.

Using a common restraint stress paradigm, we previously reported its effects on HPG axis at the level of gene transcription (10). To isolate the role of elevated CORT in causing these changes, we experimentally manipulated circulating CORT concentrations to mimic those experienced by our subjects during restraint stress. We identified genes that significantly altered their activity in response to both CORT-manipulated and restraint stress-treated groups as compared to their respective controls. We report activity changes in these genes as being indicative of the transcriptomic response to 30 min of restraint stress explained by elevated circulating CORT concentrations.

### Hypothalamus

We did not identify any hypothalamic genes in either sex that differentially expressed as a result of both restraint stress and elevated CORT. Exogenous CORT administration fails to elicit the typical patterns of sensory integration in the hypothalamus and pituitary that result in the activation of pathways associated with fear, metabolism, anxiety, and stress. Based on this assumption and our understanding of HPA axis function (both activation and negative feedback) and HPG function, we selected *a priori* 47 candidate genes for targeted analyses with relatively small effect sizes. Elevated CORT concentrations during our restraint stress treatment increased expression of prolactin receptor (*PRLR)* in males and decreased expression of Type II iodothyronine deiodinase (*DIO2)* in females. Among a myriad of other functions, prolactin also has a known role in immune and reproductive function. Increased expression of *PRLR* in the brain may act as a neurotransmitter or neuromodulator and may influence reproduction, metabolism, and/or nervous system function (23). Exposure to short-term stressors may also increase prolactin, though increases in circulating levels occurs predominately in mammals whereas in birds prolactin levels tend to be unaffected or decrease following a stressor during breeding (1, 7, 24). Administration of exogenous prolactin increased food intake, increased negative feedback of *PRL*, had antigonadotrophic effects, and reduced gonadal mass (25–29). Prolactin may act to inhibit reproduction by suppressing GnRH in breeding birds. Increased prolactin in birds has been associated with decreased estradiol and ovarian regression in females and reduced gonadal growth in males (27). Because circulating prolactin may rise following a stressor, inhibition of reproduction by prolactin may be another means that birds can adjust to their local environment and potentially reduce the energetic costs of maintaining their reproductive state when the environment is unsuitable for reproduction or to avoid an unsuccessful breeding attempt. Evidence in rats suggests that prolactin may inhibit the HPA reactivity to stress where suppression of *PRLR* increased ACTH secretion; it’s not clear if a similar effect occurs in birds (reviewed in (30–32). Dio2 converts thyroxine (T4) into triiodothyronine (T3) and stimulates metabolism. In the hypothalamus, Dio2 serves as part of a negative feedback loop on thyroid releasing hormone (33). Dio2 also plays a role in activating the reproductive axis by stimulating the production of triiodothyronine (T3) and promoting the release of gonadotropin releasing hormone (34).

An alternative, albeit non-exclusive reason for the lack of a hypothalamic transcriptomic response to exogenous CORT may be due to its heterogeneity. The avian hypothalamus contains 19 nuclei, each characterized by form and function (35). A result of this may be signal dilution, which could obstruct our identification of specific CORT-responsive substrates characteristic of the hypothalamic transcriptomic response. While this is a possibility, it is more likely that CORT treatment simply bypasses the primary action of the hypothalamus in the stress response.

### Pituitary

**Figure 7:**
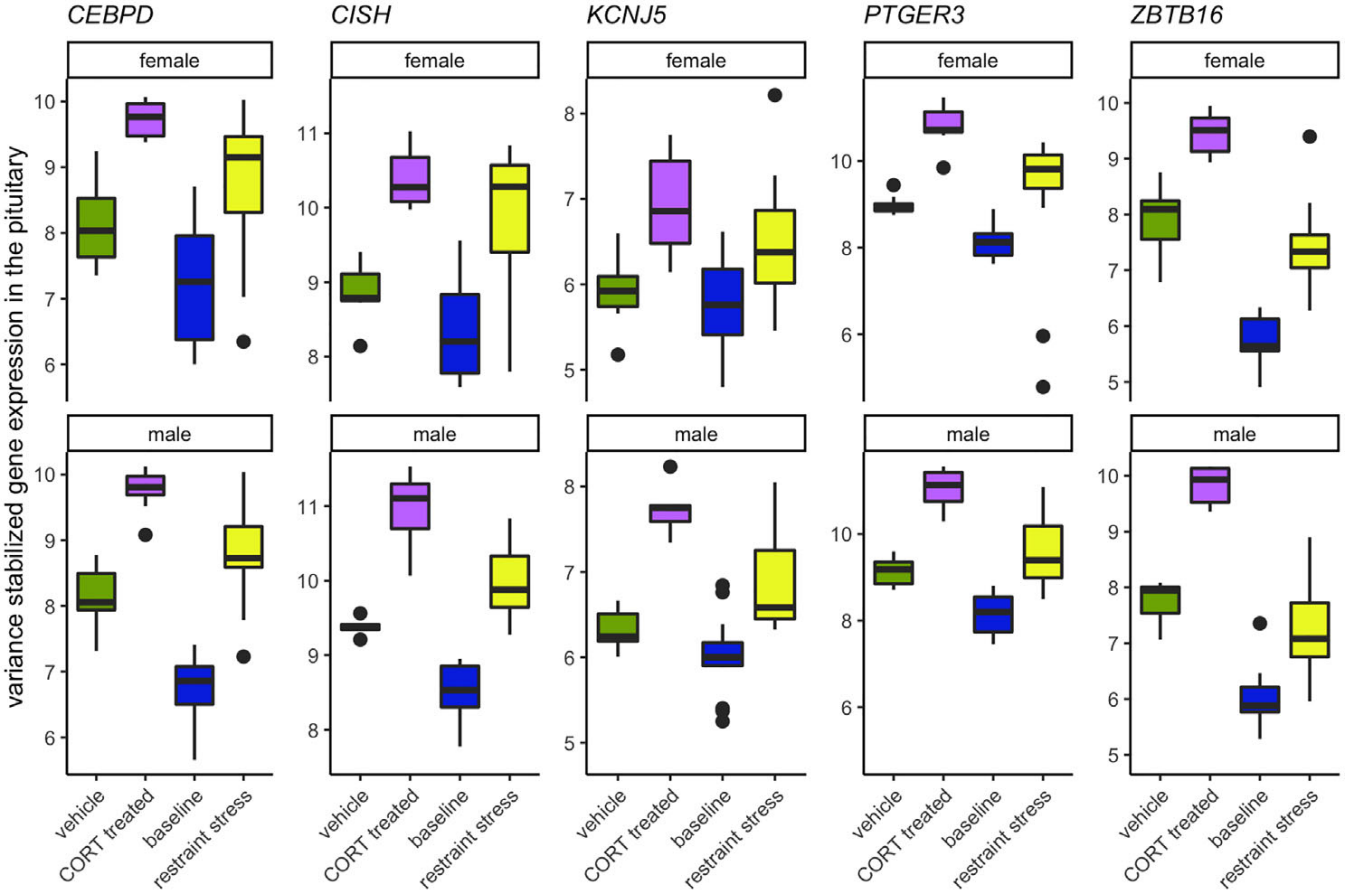
Genes that increase in response to both CORT and stress in both males and females. The elevation of CORT during the stress response resulted in the differential expression in both sexes of *KCNJ5*, *CISH*, *PTGER3*, *CEBPD*, and *ZBTB16* in the pituitary.

Elevated CORT during the stress response drives the differential expression of five genes in both sexes, all found in the pituitary: *KCNJ5*, *CISH*, *PTGER3*, *CEBPD*, and *ZBTB16* (**Figure 7**; **SI 1: Table 1**). The rapid action properties of the immediate early gene *CISH*, genes that code for transcription factors, *CEBPD* and *ZBTB16*, or with a glucocorticoid response element (GRE) on its promotor region, *KCNJ5*, suggest their potential role in early responsiveness to the CORT signal of the stress response. *PTGER3* and *CEBPD* also encode proteins that regulate prostaglandin E2 (PGE_2_). PGE_2_ is a vasodilator and immunomodulator, and thus a change in *PTGER3* and *CEBPD* may be acting to suppress immune function in response to CORT. The presence of GREs in these shared genes and several sex-specific genes suggests a mechanism by which glucocorticoids could bind and quickly induce gene transcription.

A common functional theme that emerged was the role that the products of these 5 genes play in the inflammatory immune response (*CEBPD*, *CISH*, *PTGER3*) and in the modulation of prolactin (*CEBPD, CISH*, *KCNJ5*). The role of CORT in immune and inflammatory responses has been relatively well known and are mediated by GR activation (2, 36). In brief, CORT can both activate and suppress immune function through multiple pathways (reviewed in (2, 37). However, much less in known about the role CORT plays in influencing prolactin expression or signaling pathways. While prolactin is a critical component in the initiation and mediation of aspects of reproduction and parental care, it also plays a role in the immune response, as well as the inhibition of the HPA axis (30, 31, 38). Increases in circulating CORT following a short-term stressor can result in decreased circulating prolactin in breeding birds suggesting a prolactin-stress response (7). Functional tests of these genes and their effect on reproduction, parental care, and immune function during a stress response will help to further elucidate their role.

We also isolated sex-specific effects of elevated CORT in the pituitary during the stress response. We discovered that CORT drives the differential expression of 8 genes in males (**SI 1: Table 2**) and 77 genes in females (**SI 1: Table 3**). Only three genes, *KLP9*, *PVALB*, and *SLAINL*, differentially expressed uniquely in the male pituitary. *KLF9* and *PVALB* are associated with cell and endocrine signaling and both have known responses to stress or CORT (see **SI 1: Table 2**) while the function of *SLAINL* is currently unknown. In comparison, we observed much more reactivity in female pituitary transcription (**Table 3**), with a general functional theme of their actions being related to myelination. Increased myelination and oligodendrogenesis has been reported in the hippocampus in response to exogenous glucocorticoids and immobilization stress, where it may modify the function of this tissue by decreasing neurogenesis and changing the hippocampal structure by increasing its white matter (39). It’s unclear if a similar function of increasing oligodendrogenesis and decreasing neurogenesis occurs in the pituitary, perhaps in the pars nervosa, but currently the function of increased myelination in this tissue is unknown. Another common functional theme that emerged in the pituitary included cell transport and signaling, which may be related to endocrine signaling, secretion, and feedback. Finally, we observed commonalities in gene function regarding the role of their products in immune responsiveness and as an antioxidant or mediator of oxidative stress. Transcription of genes associated with immune function or oxidative damage may, in the face of elevated CORT during a stress response, serve to modulate antioxidant activity (e.g., *APOA1*, *APOD*, *HEBP2*) or immune function (e.g., *CEBPD*, *CREB5*, *NINJ2*, *NT5E*, *TSC22D3*); however, immune actions of these differentially expressing genes in the pituitary is unclear with some acting to increase immune responsiveness (*CEBPD, NINJ2*) and others acting to inhibit it (e.g., *NT5E*, *TSC22D3*) (**SI 1: Table 3**). We can only speculate at this time as to why the female pituitary is more responsive to the CORT signal during the acute stress response. In general, the HPG transcriptomic stress response is more pronounced in females as compared to males (10). This may be because selection has favored increased stress responsiveness in females as compared to males. Females are, at least initially, more energetically invested in reproduction because they lay energy rich eggs and spend more time contact-incubating the clutch than males. A loss of a clutch or brood as a result of a stress-inducing perturbation would result in higher losses for females compared to males. As such, there is a clear selective advantage for females to be responsive to stressful conditions and respond by delaying the onset of breeding.

### Gonads

We identified the extent to which the gonadal transcriptomic response to 30 min of restraint stress could be explained by an increase in circulating CORT. In females, elevated CORT resulted in the differential expression of 28 genes during restraint stress (**SI 1: Table 4**), while in males, no changes were observed. Commonalities in functionality of genes that altered their expression within the ovaries included cell signaling and transport, immune response, and cell growth and proliferation. Future research is needed to further uncover the function of these gene products within the ovaries in response to stress.

### Non-CORT Mediated Stress-Responsive Genes

Historically, CORT has been a large focus of mechanistic inquiry behind the study of stress-induced reproductive dysfunction. Because of this, research on the influential role of CORT on the HPG axis has predominated much of the stress and reproductive biology literature, potentially overshadowing other influential mediators. We discovered 1567 non-CORT mediated genes in male and female rock doves that differentially expressed in hypothalamic, pituitary, and gonadal tissues in response to restraint stress. Although increased circulation of CORT, and glucocorticoids in general, occur in response to a variety of stressors, their elevation should not be synonymous with “stress”, as they are involved in a variety of other functions and serve as only one component of complex physiological stress responses (40). Our findings support this notion.

Potential non-CORT drivers of observed changes in gene activity in response to stress include upstream activation of the sympathetic nervous system, the hippocampus, HPA, and HPT (hypothalamus-pituitary-thyroid) axes. An example of one gene of interest that is associated with limbic activation of the stress response is *CCK* (cholecystokinin). Because of the paucity of studies of the effects of CCK on birds, the studies referenced here were conducted in mammals. CCK is an enteroendocrine hormone associated with hunger and anxiety (41, 42). In female rock doves exposed to restraint stress, *CCK* increases in the pituitary but does not respond to exogenous CORT treatment. Acute and chronic stress in the paraventricular nucleus (PVN) of the hypothalamus has been associated with increased expression of *CCK* (42), which may act as a neurotransmitter or neuromodulator in the brain (43) and interact with multiple neurotransmitter systems [e.g., dopaminergic, serotonergic, GABAergic; (42)]. CCK may also interact with the HPA axis, including corticotropin-releasing factor (CRF), to modulate stress-related physiological responses and behaviors in mammals (42). Its predominance throughout the brain suggests a role in the interaction between neurotransmitter systems, the HPA axis, and the limbic system during stress (42, 43). Thus, the function of *CCK* as a neuromodulator and in the stress response could have wide-ranging physiological and endocrinological impacts.

There also exists the potential for external influence of the HPG axis from other bodily tissues and systems that are responsive to stress. During the stress response, the HPA axis receives signals from the sympathetic nervous system and the limbic system. These signals then interact with the HPA and HPT axes to promote individual survival, often at the cost of reproductive function (1). Theoretically, exogenous administration of CORT should bypass these earliest stages of the stress response (1, 44, 45). Without input from the limbic system, upstream neuroendocrine and endocrine signaling does not activate the hypothalamic and pituitary stress response. Early CORT-independent responders to stress like catecholamines (e.g., epinephrine, norepinephrine, dopamine) are related to an increase in glucose metabolism, thereby making energy available to tissues during and after exposure to a stressor (1). These then have their own unique actions and interactions on or with tissues within the HPA, HPT, and HPG axes. Thus, the differential transcriptomic response of the HPG axis to restraint stress as compared to exogenous CORT treatment could be indicative of a lack of input from the limbic system and HPA axis prior to the synthesis of CORT. In addition, CORT-independent endocrine cascades activated by the stress response can also act directly on the HPT axis, influencing its role in regulating metabolism (33). In turn, metabolic rate can determine resource mobilization with the potential to inhibit the function of the reproductive system.

Finally, due to the dynamic and transient nature of gene transcription, translation, and physiological feedback mechanisms, we emphasize that this study provides an informative snapshot of gene transcription 30 min after exposure to a stimuli (CORT or restraint stress). It is likely that we would observe differential gene transcription at other time points. Future studies of this nature conducted along a temporal gradient will increase the resolution of our understanding of the dynamic genomic transcription and translation landscape.

### Conclusion

We report the causal and sex-typical effects of elevated CORT on the HPG stress response of the rock dove at the level of the transcriptome. We offer an extensive genomic and theoretical foundation on which to innovate the study of stress-induced reproductive dysfunction, offering novel gene targets to spur new lines of investigation and gene therapy development.

Our results suggest that elevated circulating CORT concentrations are not responsible for the majority of transcriptional changes observed in the reproductive axis following exposure of 30 minutes of restraint stress. Studies investigating the role of glucocorticoids, like CORT, in the stress response predominate much of stress biology literature. For example, a PubMed search conducted on November 21, 2020 using the search terms “stress” with “corticosterone” or “cortisol” resulted in 13,839 hits and 21,602 hits, respectively. Searches for other stress-responsive genes we have identified in this and previous (10) studies, such as “*KCNJ5”*, “*PTGER3*”, or “*CISH*”, resulted in 7, 12, and 5 hits, respectively, when associated with the search term “stress”. It may be time to shift emphasis from studying the role of this one hormone to investigating the roles of others in order to transcend our comprehension of stress and reproductive system interactions.

## Data Availability Statement

The datasets presented in this study can be found in online repositories. The names of the repository/repositories and accession number(s) can be found below: https://www.ebi.ac.uk/ena, PRJEB28645.

## Ethics Statement

The animal study was reviewed and approved by UC Davis Institutional Animal Care and Use Committee.

## Author Contributions

RC conceived the idea and developed the experimental design. JK and MM provided additional logistical advice. AB validated the CORT dosage. VF and AB conducted the data collection, SA and AB processed the tissues and biopsied hypothalami, and AL completed the RNA extraction and library preparation prior to sequencing. AL, SA, TH, RH, and MM conducted data analyses. SA drafted the manuscript, and RC edited the manuscript. All authors contributed input to the document. All authors contributed to the article and approved the submitted version. We thank two anonymous reviewers for their comments and suggestions.

## Funding

This work was funded by NSF IOS 1455960 (to RC and MM).

## Conflict of Interest

The authors declare that the research was conducted in the absence of any commercial or financial relationships that could be construed as a potential conflict of interest.
