# Supplementary material for: Isolating the Role of Corticosterone in the Hypothalamic-Pituitary-Gonadal Transcriptomic Stress Response
Source: Front Endocrinol (Lausanne). 2021 Jun 2;12:632060. doi: 10.3389/fendo.2021.632060 (PMC8207517; doi:10.3389/fendo.2021.632060)
Supplement: Supplementary file 1 [file DataSheet_1.docx]

**Supplemental Information 1: CORT and Restraint Stress.**

Genes that differentially expressed in response to CORT and restraint stress by tissue and sex. Each table contains differentially expressed genes that were found in both CORT and restraint stress treatments, which are either sex-specific or overlapping across sex. We report log fold change (logFC) and false discovery rate (FDR<0.01). Upregulated genes are highlighted in red and downregulated genes are blue.

**Table 1** Differential gene expression (DGE) shared by both sexes during the stress response due to elevated circulating CORT concentrations. Log fold change (logFC), false discovery rate (FDR) and gene function, in brief, are reported. All identified genes increased in expression in response to treatment (indicated by a negative logFC). All DGE occurred in the pituitary

| **Gene** | **Gene Name** | **Reported Function** | **CORT-treated** | | | | **Restraint stress** | | | |
| --- | --- | --- | --- | --- | --- | --- | --- | --- | --- | --- |
|  |  |  | **Female** | | **Male** | | **Female** | | **Male** | |
|  |  |  | **logFC** | **FDR** | **logFC** | **FDR** | **logFC** | **FDR** | **logFC** | **FDR** |
| *KCNJ5* | Potassium voltage-gated channel subfamily J member 5 | *KCNJ5*, also known as *GIRK4,* is a G protein regulated inward-rectifying potassium channel upregulates in response to CORT and restraint stress. *KCNJ5* is under the regulation of CORT signaling via a glucocorticoid response element (GRE) on its promoter region (1). GIRK channels can influence the resting membrane potential of cells by affecting the flow of potassium into the cell. Cells where *KCNJ5* is upregulated are more difficult to activate. Site-specific information is required to understand specific actions of corticosterone on GIRK signaling (1). In the hippocampus, neuronal exposure to corticosterone results in selective activation of ligand gated receptors through GIRK1 and 2 pathways (1). The GIRK pathway can also act as a cellular effector of dopaminergic action on lactotrophs in the anterior pituitary (2,3). Based on this action, it is feasible that upregulated *KCNJ5* may decrease prolactin, via its action on lactotrophs, following acute exposure to CORT or a stressor. Prolactin is a hormone critical for supporting parental care and immune function in birds (4). Our work did not find that *PRL* was differentially expressed in the pituitary of either treatment group though it upregulates in the male hypothalamus of CORT-treated birds. *KCNJ5* can also influence biochemical processes associated with aldosterone production thereby influencing electrolyte balance (5). Due to the influence of *KCNJ5* and GIRK pathways in the hypothalamus and prolactin in the pituitary, this gene could be a potential means by which stress affects reproduction. | -1.7 | 4.09E-06 | -1.9 | 2.70E-05 | -1.6 | 4.09E-03 | -1.5 | 7.27E-03 |
| *CISH* | Cytokine-inducible SH2-containing protein | *CISH* is another shared gene that upregulates in response to both restraint stress and CORT treatments. *CISH* is an immediate early gene that is involved in negative feedback of the Jak2-Stat5 pathway (6,7). The CISH protein competitively binds to phosphorylated prolactin receptors (PRLr), which attenuates Stat5 binding and subsequent downstream pathway signaling (49,50). This action is one way that CISH may inhibit prolactin transcription and downstream events of the Jak-Stat pathway. Evidence also suggests that cytokines are important cell-signaling molecules that play a role in the inflammatory response (9). Thus, if increased CISH activity is indicative of decreased cytokine activity or attenuation of PRLr binding, this could be a possible mechanism by which stress could influence both immune function and parental care. | -1.6 | 3.72E-07 | -1.7 | 2.63E-05 | -1.6 | 1.91E-10 | -1.8 | 3.16E-10 |
| *PTGER3* | Prostaglandin E receptor 3 | *PTGER3* increased in expression in response to both restraint stress and CORT-treatments. *PTGER3* encodes a G-protein-coupled receptor, EP3, that binds to its ligand, PGE2. The binding of EP3 to PGE2 results in the activation of an inhibitory G protein that reduces adenylyl cyclase activity and causes a decline in cellular cAMP.  Prostaglandins have been linked to inflammation-induced activation of the HPA axis and act as an important immunoregulator (10–12). PGE_2_ binds to EP1 and EP3, which promote an ACTH response (10). Recall that ACTH is synthesized in the pituitary and travels through the bloodstream where it binds to melanocortin 2 receptors in the adrenals and promotes CORT synthesis. Cytokines may also induce prostaglandin synthesis (PGE_2_) and EP3 activation of the HPA axis (10). EP3’s importance in inflammation-induced HPA activation was shown by a study that used addressed how EP3 knockout mice responded to an induced fever (by injecting pyrogen; ,12). EP3 ^-/-^ mice did not develop a fever following treatment with pyrogen whereas controls did (12). This result suggests that EP3 activates the HPA axis in response to illness (12). Activation of the HPA via EP3 also includes vasodilation of the skin and increased metabolism of brown fat (10). *PTGER3* may be another means by which the HPA axis is activated, J7and critically, *PTGER3* appears to be a means that the HPA axis primes itself for potential injury or infection following exposure to a stressor. | -2 | 2.19E-07 | -2 | 2.70E-05 | -1.5 | 7.50E-03 | -1.8 | 7.91E-04 |
| *CEBPD* | CCAAT enhancer binding protein delta | *CEBPD* upregulates in response to CORT and restraint stress. This gene encodes a CCAAT/enhancer-binding protein that acts as transcription factor and responds to glucocorticoids (13). These proteins act in metabolism, adipogenesis, immune and inflammatory responses, and PRL regulation (13,14). Tong et al. (14) suggest that PRL expression and lactotroph cell proliferation in the pituitary are linked: *CEBPD* plays a key role in coordinating their actions. Forced expression of *CEBPD* suppressed PRL expression in PRL-secreting cells and lactotroph proliferation in rats by binding to the PRL promoter (14). *CEBPD* may also be activated by a number of inflammatory factors such as PGE2 (13). Transcription of *CEBPD* following an inflammatory stimuli can be activated by a number of signaling pathways including JAK (13). Thus, upregulation of this gene may increase inflammatory responsiveness and suppress prolactin synthesis in response to a stressor or environmental perturbation. This gene is another potential target for understanding the role of CORT on the inflammatory response and suppression of reproduction. | -1.6 | 1.25E-06 | -1.7 | 3.50E-05 | -1.9 | 1.73E-05 | -2.6 | 3.10E-09 |
| *ZBTB16* | Zinc finger and BTB domain containing 16 | *ZBTB16* upregulates in the pituitary of males and females exposed to restraint stress and CORT. It is a transcription factor that generally acts as a transcriptional repressor, downregulating gene transcription through recruitment of other transcriptional co-repressors and histone deacetylases that modify chromatin (15,16). This protein has been implicated in reducing cell proliferation and increasing cell death via apoptosis in various cancer cell lines and during development (16). Glucocorticoids have been shown to upregulate *ZBTB16* in the brain of mice and cancer cell lines (17,18). While no known study has examined *ZBTB16* in the pituitary, it may possibly play a role in the negative feedback of CORT on pituitary gene expression and cell proliferation. For example, *ZBTB16* may repress transcription and recruit epigenetic mechanisms, to downregulate genes associated with HPA axis activity. Further, *ZBTB16* may inhibit cell growth and proliferation of corticotrophs in the pituitary. Glucocorticoids have been shown to suppress growth of corticotrophic tumors (19) and have generally inhibitory effects on DNA synthesis in the pituitary (20), possibly through a mechanism including *ZBTB16*. Taken together, these results suggest *ZBTB16* may play a role in negative feedback of the HPA axis by affecting corticotroph transcription and cell proliferation. | -1.7 | 1.25E-06 | -2.3 | 9.47E-09 | -2.9 | 5.57E-08 | -2.2 | 2.95E-05 |

**Table 2:** Differential gene expression (DGE) in the male pituitary during the stress response due to elevated circulating CORT concentrations. Red indicates upregulation while blue indicates downregulation.

| **Entrez ID** | **Gene** | **Gene Name** | **Function** | **CORT** | | **Restraint stress** | |
| --- | --- | --- | --- | --- | --- | --- | --- |
|  |  |  |  | **logFC** | **FDR** | **logFC** | **FDR** |
| 107052707 | *CEBPD* | CCAAT enhancer binding protein delta | Described in Table 1 | -1.73 | 3.50E-05 | -2.57 | 3.10E-09 |
| 395335 | *CISH* | cytokine inducible SH2 containing protein | Described in Table 1 | -1.70 | 2.63E-05 | -1.76 | 3.16E-10 |
| 395925 | *KCNJ5* | potassium voltage-gated channel subfamily J member 5 | Described in Table 1 | -1.92 | 2.70E-05 | -1.48 | 7.27E-03 |
| 770238 | *KLF9* | Kruppel like factor 9 | Krüppel-like factors are a group of highly conserved genes in vertebrates that act as immediate early genes or hormone mediating transcription factors. They can be direct transcription factors, cofactors/accessory transcription factors, or can mediate nuclear receptor genes to regulate hormone function (21). *KLF9* responds independently or synergistically to thyroid hormones and glucocorticoids in the brains of mice and frogs while in the uterus and ovaries it responds to progesterone and estradiol (21). CORT acts on *KLF9* via a GRE in its promoter region (21). This gene influences the structure and differentiation of neurons and axons (21,22). *KLF9* influenced dendritic spine growth in the hippocampus of mice that were exposed to chronic stress (23). In a study of juvenile frogs, shaking/confinement and intraperitoneal injections of CORT increased *KLF9* mRNA expression in the brain (22). Exposing frogs to a stressor and, subsequently, to a GR antagonist decreased expression of *KLF9* while treatment with a mineralocorticoid antagonist had no effect (22). This work suggests that CORT and GR act to regulate the actions of *KLF9* (21). Little work has been done on this gene in the pituitary, but as an immediate early gene that appears to respond to thyroid hormones, CORT, and other steroid hormones, it may also act to regulate these hormones in the pituitary. | -1.17 | 4.13E-03 | -0.87 | 3.66E-03 |
| 429116 | *PTGER3* | prostaglandin E receptor 3 | Reported in Table 1 | -2.02 | 2.70E-05 | -1.82 | 7.91E-04 |
| 396459 | *PVALB* | parvalbumin | *PVALB* encodes parvalbumin and regulates Ca^2+^ and Mg^+^ binding activity (24). Cells containing parvalbumin can act as GABAergic interneurons, which have been found to be associated with the stress response in the hippocampus (24). In response to chronic high CORT levels, parvalbumin containing cells decreased in the hippocampus of rodents; however, in response to acute stress, Pv-containing cells increased (24). The response of *PVALB* to glucocorticoids or restraint stress in the pituitary has not been studied. | 2.92 | 2.60E-04 | 3.49 | 9.54E-05 |
| 100859468 | *SLAINL* | SLAIN motif-containing protein-like | Unknown | -1.15 | 1.12E-04 | -1.12 | 4.92E-03 |
| 419759 | *ZBTB16* | zinc finger and BTB domain containing 16 | Described in Table 1 | -2.31 | 9.47E-09 | -2.16 | 2.95E-05 |

**Table 3**: Differential gene expression (DGE) in the pituitary in females during the stress response due to elevated circulating CORT concentrations. Red indicates upregulation while blue indicates downregulation.

| **Entrez ID** | **Gene** | **Gene Name** | **Reported function** | **CORT** | | **Restraint stress** | |
| --- | --- | --- | --- | --- | --- | --- | --- |
|  |  |  |  | **logFC** | **FDR** | **logFC** | **FDR** |
| 431627 | *ACOT12* | acyl-CoA thioesterase 12 | *ACOT12* encodes acyl-coenzyme A thioesterase 12 (also known as StAR-related lipid transfer protein 15). It is involved in a number of functions including acetyle-CoA hydralase activity (GO:0003986) (25). | -2.53 | 7.93E-03 | -2.17 | 4.96E-05 |
| 420738 | *ANLN* | anillin actin binding protein | *ANLN* encodes an actin-binding protein that contributes to cytokinesis and cell growth and migration (25). | -4.89 | 1.71E-06 | -6.33 | 4.03E-11 |
| 419316 | *APCDD1L* | APC down-regulated 1 like | *APCDD1L* encodes a protein of unknown function. | -2.79 | 2.19E-07 | -1.43 | 4.75E-03 |
| 396536 | *APOA1* | apolipoprotein A1 | *APOA1* encodes the protein, alolipoprotein A-I, which is associated with high-density lipoprotein and acts in the transport of cholesterol and phospholipid (26). APOA1 also functions as an anti-inflammatory and antioxidant (26). | -2.29 | 6.29E-03 | -4.13 | 3.08E-07 |
| 424893 | *APOD* | apolipoprotein D | *APOD* encodes apolipoprotein D, which can bind to arachidonic acid, progesterone, retinol, cholesterol, among others (27). It appears to be a stress responsive gene that acts in mediating oxidative stress (27). | -3.58 | 1.22E-03 | -6.76 | 5.12E-10 |
| 417431 | *APOH* | apolipoprotein H | *APOH* encodes apolipoprotein H, which is implicated in a number of physiological processes (e.g., lipoprotein metabolism and coagulation) though its specific function is unknown (25). | -5.67 | 3.72E-07 | -6.98 | 1.48E-09 |
| 769889 | *APOLD1* | apolipoprotein L domain containing 1 | *APOLD1* is a stress-responsive immediate early gene that appears to be regulated by beta-adrenergic activity (28). It functions in angiogenesis (29). | -1.96 | 4.77E-05 | -2.79 | 1.26E-06 |
| 421088 | *AQP4* | aquaporin 4 | *AQP4* encodes an aquaporin protein that functions in water homeostasis (25). | -2.89 | 2.32E-04 | -4.10 | 2.42E-08 |
| 421369 | *ATF3* | activating transcription factor 3 | *ATF3* encodes a member of the activating transcription factor/cyclic adenosine monophosphate(cAMP)-response element binding (CREB) protein family (30). It is associated with cell stress and can act as a transcriptional activator or repressor, depending on context (30). | -1.82 | 2.50E-04 | -2.96 | 2.76E-08 |
| 422966 | *C5H11ORF9* | chromosome 5 open reading frame, human C11orf9 | *C5H11ORF9* or the myelin regulatory factor (*MYRF*) in humans encodes a transcription factor associated with a key regulator of myelination and may directly stimulate gene expression of myelin (31). | -4.23 | 6.29E-05 | -5.59 | 8.78E-11 |
| 423959 | *C6H10ORF90* | chromosome 6 open reading frame, human C10orf90 | *C6H10ORF90* is a p53 responsive gene that acts in tumor suppression (32). It is unclear what role this gene is playing here. | -2.14 | 8.04E-03 | -4.13 | 2.74E-10 |
| 416927 | *CA15L* | carbonic anhydrase 15-like | *CA15L* encodes a protein of unknown function. | -5.56 | 2.97E-07 | -5.85 | 9.73E-06 |
| 421029 | *CDH19* | cadherin 19 | *CDH19* encodes a calcium dependent cell-cell adhesion protein (25). | -3.91 | 4.48E-05 | -4.50 | 1.07E-09 |
| 107052707 | *CEBPD* | CCAAT enhancer binding protein delta | Described in Table 1 | -1.63 | 1.25E-06 | -1.89 | 1.73E-05 |
| 395335 | *CISH* | cytokine inducible SH2 containing protein | Described in Table 1 | -1.64 | 3.72E-07 | -1.81 | 1.91E-10 |
| 395399 | *CITED2* | Cbp/p300 interacting transactivator with Glu/Asp rich carboxy-terminal domain 2 | *CITED2* encodes a protein that competitively binds to hypoxia-inducible factor 1-alpha (*HIF1A*) thereby inhibiting expression of *HIF1A*-induced genes (25). | -1.38 | 7.43E-07 | -0.90 | 7.93E-03 |
| 395921 | *CNP* | 2',3'-cyclic nucleotide 3' phosphodiesterase | *CNP* encodes a protein that encodes a precursor of *NPCC* (C-type natriuretic peptide). NPCC is associated with vasodilation and natriuresis. (25) | -3.15 | 1.69E-05 | -5.09 | 4.87E-12 |
| 428435 | *CREB5* | cAMP responsive element binding protein 5 | *CREB5* encodes a cAMP response element (CRE) binding protein (33). CREB5 regulates cell growth, proliferation, and differentiation, and may be involved in the immune response (33). Recent work showed that hypo-methylation and upregulation of *CREB5* in decidua was associated with recurrent pregnancy loss in humans (33). | -1.97 | 7.07E-03 | -3.97 | 4.16E-07 |
| 421422 | *DAAM2* | dishevelled associated activator of morphogenesis 2 | *DAAM2* helps regulate the planar cell polarity pathway, a Wnt signaling pathway (34). *Daam2* appears to inhibit the differentiation of oligodendrocytes thereby impacting myelination (via the PIP5K-PIP2 axis; (34). | -1.50 | 7.69E-03 | -2.71 | 1.99E-08 |
| 107053650 | *DDIT4* | DNA damage inducible transcript 4 | *DDIT4* is a stress responsive gene that suppresses mTor (Mechanistic Target of Rapamycin) pathways (35,36). Upregulation of *DDIT4* occurs following cellular damage associated with a number of stressors (35,36). *DDIT4* also responds to the presence of glucocorticoids via a GRE-like element on its promoter (36). Upregulation of *DDIT4* results in the suppression of the mTOR pathway, which has been associated with reduced cell growth and plasticity in several tissues and an increase in cell apoptosis (36).  Most of the research on this gene has focused on the hippocampus– its role in the pituitary has not been well-studied. | -1.49 | 1.21E-08 | -2.75 | 9.40E-08 |
| 425241 | *DIRAS2* | DIRAS family GTPase 2 | *DIRAS2* likely acts in GTP binding (GO:0005525) and GTPase activity (GO:0003924). | -1.58 | 1.71E-04 | -1.41 | 4.23E-03 |
| 422551 | *ENPP6* | ectonucleotide pyrophosphatase/phosphodiesterase 6 | *ENPP6* is thought to be involved in extracellular nucleotide metabolism regulation and myelination (25). | -2.37 | 2.03E-03 | -2.78 | 3.56E-06 |
| 771826 | *ERMN* | ermin | *ERMN* encodes a protein that is associated with myelination and mature nerves (37). | -5.22 | 1.69E-05 | -4.37 | 2.65E-08 |
| 426690 | *ETNK2* | ethanolamine kinase 2 | *ETNK2* is associated with early catalysis of the ethanolamine pathway (25). | -2.64 | 7.83E-04 | -4.16 | 1.90E-08 |
| 415687 | *FA2H* | fatty acid 2-hydroxylase | *FA2H* encodes the enzyme, fatty acid 2-hydroxylase (25). It may be involved in fatty acid modification and myelin maintenance (25). | -4.68 | 1.33E-05 | -5.36 | 1.78E-10 |
| 422413 | *FAM198B* | family with sequence similarity 198 member B | *FAM198B* is predominately expressed in the adrenal and ovary (25). The function of this gene is currently unknown. | -3.14 | 3.01E-03 | -2.93 | 3.72E-03 |
| 419084 | *GDPD4* | glycerophosphodiester phosphodiesterase domain containing 4 | Little information is known about *GDPD4*, but it is involved in glycerophosphodiester phosphodiesterase activity (GO:0008889), metal ion binding (GO:0046872), and the lipid metabolic process (GO:0006629) (25). | -2.29 | 4.25E-04 | -2.16 | 2.78E-03 |
| 419969 | *GFAP* | glial fibrillary acidic protein | *GFAP* encodes glial fibrillary acidic protein, which is an intermediate filament protein found in a number of cell types in the CNS (e.g., astrocytes) (25). | -5.98 | 8.37E-05 | -7.93 | 9.40E-12 |
| 420397 | *GJC2* | gap junction protein gamma 2 | *GJC2* encodes a gap junction protein that is involved in myelination (25). | -1.65 | 4.42E-03 | -2.93 | 4.70E-08 |
| 427523 | *GLDN* | gliomedin | *GLDN* encodes an olfactomedin-like and collagen-like domain protein that can occur in both transmembrane and secreted forms. It plays a role in the formation of the nodes of Ranvier in the peripheral nervous system. (25) | -3.10 | 9.43E-05 | -1.43 | 8.56E-03 |
| 396489 | *GLUL* | glutamate-ammonia ligase | *GLUL* encodes a member of the glutamine synthetase family, which plays a role in the catalysis of glutamine synthesis and glutamate detoxification, cell signaling and proliferation, and acid-base homeostasis (25). | -1.87 | 1.10E-03 | -4.03 | 7.05E-11 |
| 417748 | *GPR37* | G protein-coupled receptor 37 | *GPCR* and *GPCRL1* are associated with the regulation of neuronal and glial physiology and modulate dopaminergic neurotransmission(38). These receptors are bound by prosaposin and prosaptide, which stimulates ERK phosphorylation and protect against cellular stress (38). Prosaposin has neuroprotective and glioprotective action, which is likely mediated by these receptors (38). | -3.32 | 2.28E-03 | -4.80 | 2.76E-08 |
| 421176 | *GPR37L1* | G protein-coupled receptor 37 like 1 | *GPCR* and *GPCRL1* are associated with the regulation of neuronal and glial physiology and modulate dopaminergic neurotransmission (38). These receptors are bound by prosaposin and prosaptide, which stimulates ERK phosphorylation and protect against cellular stress (38). Prosaposin has neuroprotective and glioprotective action, which is likely mediated by these receptors (38). | -3.92 | 1.29E-03 | -5.14 | 2.43E-07 |
| 100859224 | *GPR62* | G protein-coupled receptor 62 | Little is known about *GPCR62*, but this gene encodes a protein that may regulate cAMP production (39). | -5.86 | 6.93E-05 | -6.58 | 1.94E-09 |
| 425975 | *HAPLN2* | hyaluronan and proteoglycan link protein 2 | *HAPLN2* encodes a hyaluronan and proteoglycan binding link protein that is acts in the stabilization and increase of binding of four chondroitin sulfate proteoglycans core proteins and in maintaining the extracellular matrix (40). It is expressed in myelinated fiber in the brain and overexpression in rats was associated with cell death of neurons (40). | -5.40 | 3.01E-03 | -7.37 | 7.05E-11 |
| 424441 | *HEBP2* | heme binding protein 2 | *HEBP2* encodes a protein that is associated with the collapse of mitochondrial membrane potential in the cytoplasm and enhances mitochondrial membrane permeability during oxidative stress (25). | -2.54 | 1.95E-03 | -3.30 | 4.98E-10 |
| 428234 | *HEPACAM* | hepatic and glial cell adhesion molecule | *HEPACAM* encodes a protein that functions in cell motility or cell-matrix interaction (25). | -3.88 | 6.93E-05 | -4.03 | 2.55E-06 |
| 395128 | *HES4* | hes family bHLH transcription factor 4 | *HES4* is associated with the Notch signaling pathway (41). | 1.44 | 3.68E-06 | 1.40 | 3.70E-06 |
| 420476 | *JCAD* | junctional cadherin 5 associated | *JCAD* or *KIAA1462* encodes a cell-cell junction protein (25). | -1.08 | 8.23E-03 | -1.67 | 1.54E-04 |
| 427662 | *KCNJ12* | potassium voltage-gated channel subfamily J member 12 | *KCNJ12* encodes an inwardly rectifying K+ channel (25). | -3.09 | 5.26E-03 | -4.24 | 1.43E-06 |
| 395925 | *KCNJ5* | potassium voltage-gated channel subfamily J member 5 | Described in Table 1 | -1.74 | 4.09E-06 | -1.55 | 4.09E-03 |
| 422374 | *KIAA1210* | *KIAA1210* | *KIAA1210* may be a cell junction protein involved with mammalian spermiogenesis in the testes, but little else is known about its function in other tissues (42). | -1.29 | 2.34E-03 | -1.48 | 1.33E-04 |
| 395705 | *LHX2* | LIM homeobox 2 | *LHX2* functions in transcription regulation (25). | -2.91 | 1.29E-03 | -2.42 | 3.16E-03 |
| 100859848 | *LOC100859848* | CDC42 small effector protein 2-C-like | *LOC100859848* encodes a protein of unknown function. | -2.20 | 3.28E-04 | -3.26 | 3.32E-09 |
| 107050516 | *LOC107050516* | Schwann cell myelin protein-like | *LOC107050516* encodes a protein of unknown function. | -3.62 | 7.88E-06 | -4.40 | 2.00E-07 |
| 769726 | *LOC769726* | kazal-type serine protease inhibitor domain-containing protein 1-like | *LOC769726* encodes a protein of unknown function. | -5.64 | 1.15E-06 | -6.93 | 1.81E-10 |
| 396217 | *MBP* | myelin basic protein | *MPB* encodes myelin basic protein, which is a critical component of the myelin sheath of oligodendrocytes and Schwann cells in the CNS and in myelination (25). | -1.81 | 1.77E-03 | -3.92 | 4.37E-12 |
| 417737 | *MLC1* | megalencephalic leukoencephalopathy with subcortical cysts 1 | *MLC1* encodes a product of unknown function (25). | -2.15 | 9.18E-04 | -3.98 | 2.76E-08 |
| 418151 | *NINJ2* | ninjurin 2 | *NINJ2* encodes a protein of the ninjurin (for nerve injury induced) family, which is a cell surface adhesion molecule that is related to nerve injury and neurite outgrowth (43). It has also been suggested as a regulator of multiple proteins associated with inflammation *(*GDNF*,* GM-CSF*,* ICAM-1, IL-1*β*, M-CSF, TGF-*β*1, TGF-*β*3, TNF-*α*, TNF-*β*, BLC, CCL28, Fractalkine, GCP-2, I-TAC, IL-8, Lymphotactin, MIP-3*β*, MIP-3*α* and TECK) and may influence the gene expression of several associated genes (*GDNF, ICAM-1, IL-1β, M-CSF, TGF-β1, TGF-β3, BLC, CCL28, Fractalkine, GCP-2, I-TAC, IL-8, Lymphotactin, MIP-3β, MIP-3α and TECK*), as well (43). *NINJ2 and TLR4* also regulate NF-KB and c-jun pathways (43). | -5.13 | 1.15E-06 | -6.08 | 7.05E-11 |
| 421833 | *NT5E* | 5'-nucleotidase ecto | *NT5E* encodes a plasma membrane protein that can act to inhibit immune response via its generation of a adenosine (44). | -4.37 | 1.93E-04 | -3.29 | 4.39E-06 |
| 428612 | *OLIG2* | oligodendrocyte transcription factor 2 | *OLIG2* encodes a transcription factor that regulates oligodendrocyte progenitor cells, ventral neuroectodermal progenitor cell fate and chromosomal translocation (25,31). Oligodendrocytes act in CNS myelination (31). | -5.52 | 3.28E-05 | -7.78 | 1.80E-11 |
| 395334 | *OPN4-1* | photopigment melanopsin-like | *OPN4-1* is associated with melanopsin biosynthesis and may contribute to circadian entrainment in the tissue of expression (45). | -4.43 | 2.97E-03 | -5.74 | 3.54E-07 |
| 771808 | *PIPOX* | pipecolic acid and sarcosine oxidase | *PIPOX* is associated with L-pipecolate oxidase activity (GO:0050031), sarcosine oxidase activity (GO:0008115), and signaling receptor binding (GO:0005102). | -3.60 | 1.89E-03 | -5.09 | 9.15E-05 |
| 415650 | *PLLP* | plasmolipin | *PLLP* functions in myelin biosynthesis though little more is known about this protein (46). | -4.55 | 5.36E-06 | -7.10 | 4.88E-11 |
| 396214 | *PLP1* | proteolipid protein 1 | *PLP1* encodes a key component of myelin and may be associated with various functions related to myelin (25). | -8.93 | 3.52E-07 | -10.69 | 4.37E-12 |
| 420198 | *PMP2* | peripheral myelin protein 2 | *PMP2* encodes a protein that is associated with myelin sheaths in the peripheral nervous system that may act in sheath stabilization (25). | -7.36 | 2.32E-04 | -4.79 | 3.77E-04 |
| 417327 | *PMP22* | peripheral myelin protein 22 | *PMP22* encodes an integral membrane protein of myelin and the peripheral nervous system (25). | -2.66 | 6.29E-04 | -2.27 | 2.16E-07 |
| 429116 | *PTGER3* | prostaglandin E receptor 3 | Described in Table 1 | -1.99 | 2.19E-07 | -1.51 | 7.50E-03 |
| 419521 | *RASSF2* | Ras association domain family member 2 | *RASSF2* functions in cell growth inhibition, cell cycle arrest, actin cytoskeleton organization, apoptosis, suppression of transcription of NFKB, and inhibition of *MST2* activity (47). | -3.05 | 1.02E-04 | -4.17 | 9.38E-09 |
| 424038 | *S100B* | S100 calcium binding protein B | *S100B* has been linked to cell survival, proliferation and differentiation, neuromodulation, cell migration, cell morphogenesis and proliferation, and cytoskeletal dynamic, depending on its effector (48). | -4.31 | 4.90E-05 | -4.45 | 5.98E-08 |
| 424593 | *SEPP1L* | selenoprotein P2 | The function of *SEP1L* is unknown, but it may act as an extracellular antioxidant (25). | -4.12 | 5.86E-05 | -5.43 | 5.29E-09 |
| 416778 | *SEPT5* | septin 5 | *SEPT5* encodes a nucleotide binding protein that is associated with regulation of cytoskeletal organization (25). | -1.81 | 1.05E-03 | -3.32 | 7.05E-11 |
| 107049626 | *SFTPC* | SFTPC surfactant protein C | *SFTPC* encodes surfactant protein C, which is a protein required for lung function (25). It is unclear why it is differentially expressed in the female pituitary as expression is thought to be restricted to lungs (25). | -4.17 | 7.93E-03 | -6.18 | 8.09E-06 |
| 418731 | *SH3RF3* | SH3 domain containing ring finger 3 | *SH3RF3* encodes a scaffold protein associated with E3 ligase activity, which may be involved in JNK-mediated apoptosis (49). | -2.12 | 1.90E-03 | -1.86 | 1.38E-03 |
| 396089 | *SHANK3* | SH3 and multiple ankyrin repeat domains 3 | *SHANK3* is involved in synapse function, specifically as a scaffold protein involved in connecting neurotransmitter receptors and ion channels to G protein receptor signaling pathways or actin cytoskeleton (25,50). It is also involved in dendritic spine morphology (50). | -5.62 | 2.21E-04 | -7.31 | 1.24E-10 |
| 395615 | *SHH* | sonic hedgehog | *SHH* encodes a protein involved in the hedgehog pathway. Most notably associated with embryonic development, SHH could play a role in neurogenesis, and the maintenance of homeostasis and repair of neural stem cell progenitors in adult tissues (51). | 1.95 | 9.50E-03 | -2.66 | 7.57E-04 |
| 424576 | *SLC6A9* | solute carrier family 6 member 9 | *SLC6A9* encodes a protein that inhibit glycine signaling, a neurotransmitter inhibitor of the CNS (25). | -2.93 | 6.29E-05 | -4.37 | 2.29E-09 |
| 432368 | *SNAI2* | snail family transcriptional repressor 2 | *SNAI2* encodes a transcription factor that is associated with embryonic development, but it is also suggested to play a role in maintaining normal function in mature cells as it is found in most tissues (25). | 1.48 | 2.28E-03 | 1.37 | 1.41E-03 |
| 395573 | *SOX10* | SRY-box 10 | *SOX10* is a transcription factor typically associated with embryonic development. However, in adult cells, Sox10 complexes with Olig10 to activate mbp (myelin basic protein) transcription. (52) | -4.46 | 5.99E-04 | -6.84 | 6.17E-09 |
| 395483 | *SOX8* | SRY-box 8 | *SOX8* is a SRY-related HMG-box transcription factor, which is associated with embryonic development regulation and cell fate determination (25). | -2.31 | 5.01E-05 | -3.74 | 1.55E-09 |
| 419414 | *TMEM88B* | transmembrane protein 88B | *TMEM88B* encodes a protein with a currently unknown function. | -5.07 | 1.69E-04 | -5.69 | 1.55E-09 |
| 396032 | *TNNC1* | troponin C1, slow skeletal and cardiac type | *TNNC1* encodes a regulatory protein associated with muscle contraction in the actin filament (25). | -4.79 | 2.88E-05 | -5.67 | 2.00E-06 |
| 768091 | *TSC22D3* | TSC22 domain family member 3 | *TSC22D3* encodes an anti-inflammatory protein glucocorticoid-induced leucine zipper that is stimulated by glucocorticoids and interleukin-10. It functions in inhibiting inflammation and the immune system. (25) | -1.02 | 1.22E-03 | -1.65 | 1.16E-06 |
| 428900 | *TSHR* | thyroid stimulating hormone receptor | *TSHR* is a gene that transcribes the thyroid stimulating hormone receptor. The thyroid stimulating hormone receptor responds to the presence of thyroid stimulating hormone, a metabolic hormone that stimulates the thyroid to produce thyroxine (T4) and triiodothyronine (T3). Other studies have shown that glucocorticoids increase expression of *TSHR* in the pituitary (53). While this gene was upregulated, there was high variance, which suggests a trend towards increased expression of *TSHR* (Fig. 6). While pharmaceutical doses of glucocorticoids in rats can suppress circulating TSH, mRNA *TSH*, and thereby, *TSHR*, this study found that *TSHR* was upregulated (53). This result may indicate an increase in metabolism in response to glucocorticoids and restraint stress. | -3.13 | 8.29E-03 | -3.66 | 3.70E-05 |
| 100858879 | *TTYH2* | tweety family member 2 | *TTYH2* encodes a protein that influences calcium (2+)-activated large conductance chloride(-) channels (25). | -1.79 | 4.79E-03 | -3.86 | 1.72E-09 |
| 374033 | *UGT8* | UDP glycosyltransferase 8 | *UGT8* encodes 2-hydroxyacylsphingosine 1-beta-galactosyltransferase, which is associated with myelin (54). | -2.67 | 3.53E-04 | -3.50 | 2.49E-09 |
| 419759 | *ZBTB16* | zinc finger and BTB domain containing 16 | Described in Table 1 | -1.66 | 1.25E-06 | -2.86 | 5.57E-08 |

**Table 4:** Differential gene expression (DGE) in the ovaries due to elevated circulating CORT concentrations. Red indicates upregulation while blue indicates downregulation.

| **Entrez ID** | **Gene** | **Gene Name** | **Reported function** | **CORT** | | **Restraint stress** | |
| --- | --- | --- | --- | --- | --- | --- | --- |
|  |  |  |  | **logFC** | **FDR** | **logFC** | **FDR** |
| 418254 | *A2ML1* | alpha-2-macroglobulin like 1 | *A2ML1* encodes an alpha-macroglobulin member protein (25). | 3.90 | 1.71E-05 | 2.71 | 2.58E-04 |
| 373945 | *ABCA1* | ATP binding cassette subfamily A member 1 | *ABCA1* encodes a protein in the ATP-binding cassette (ABC) transporters 1 family. In mice inflammatory stress decreased expression of cholesterol mice associated with ABCA-1 (55). | -0.86 | 7.74E-03 | 3.31 | 3.78E-03 |
| 771077 | *AK9/AKD1* | adenylate kinase 9 | *AK9* is involved in nucleoside homeostasis (25). | -2.40 | 9.66E-05 | -1.53 | 1.97E-04 |
| 420744 | *BMPER* | BMP binding endothelial regulator | *BMPER* encodes a glycoprotein that directly modulates bone morphogenetic protein (a member of the transforming growth factor-beta family) signaling and has been found in the mammalian prostate and testes (25). BMPs are associated with serine/threonine kinase receptor signaling and cell proliferation (56). | -1.51 | 5.94E-03 | -1.18 | 5.02E-03 |
| 417647 | *CA4* | carbonic anhydrase 4 | *CA4* encodes carbonic anhydrase 4, a member of zinc metalloenzymes that is involved in the catalysis of reversible hydration of carbon dioxide (25). The exact function of carbonic anhydrase 4 is unknown (25). | -3.10 | 3.61E-07 | -3.40 | 3.89E-07 |
| 427515 | *CALML4* | calmodulin like 4 | Little is known about *CALML4* but GO terms suggest it functions in calcium-ion binding (GO:0005509), calcium-mediated signaling (GO:0019722), regulation of catalytic activity (GO:0050790) and spindle pole body organization (GO:0051300). | -1.88 | 9.06E-03 | -2.27 | 2.38E-03 |
| 424601 | *CCDC17* | coiled-coil domain containing 17 | Little is known about *CCDC17*, but it appears to function in protein binding (GO:0005515). | -3.05 | 4.63E-03 | -2.60 | 5.23E-04 |
| 107052707 | *CEBPD* | CCAAT enhancer binding protein delta | Described in Table 1 | -1.48 | 2.16E-05 | -1.43 | 7.08E-04 |
| 374002 | *CKMT1A* | creatine kinase, mitochondrial 1A | *CKMT1A* encodes mitochondrial creatine kinase, which acts in movement of phosphate to creatine within the mitochondria thereby functioning in cell metabolism (25). It also plays a critical role in regulating the permeability transition pore where downregulation leads to the depolarization of mitochondria and apoptosis (57). | 2.62 | 4.88E-08 | -2.08 | 1.38E-04 |
| 769188 | *FAM161A* | FAM161A, centrosomal protein | *FAM161A* encodes a protein associated with retinal photoreceptors (25). Its function here is unclear. | -1.58 | 9.02E-04 | -1.22 | 5.10E-04 |
| 428618 | *FLRT1* | fibronectin leucine rich transmembrane protein 1 | *FLRT1* encodes a fibronectin leucine rich transmembrane protein (25). | 3.94 | 8.93E-03 | -2.41 | 7.65E-04 |
| 423079 | *HPS5* | *HPS5*, biogenesis of lysosomal organelles complex 2 subunit 2 | *HPS5* encodes a protein involved in the biogenesis of melanosomes, platelet dense granules and lysosomes (25) | -2.80 | 1.07E-04 | -2.80 | 5.05E-05 |
| 422219 | *IL13RA2* | interleukin 13 receptor subunit alpha 2 | *IL12RA2* binds with high affinity to interleukin 13, but its specific function is unknown (25). | 2.08 | 3.39E-04 | -1.97 | 1.21E-03 |
| 418840 | *LACC1* | laccase domain containing 1 | *LACC1* encodes an oxidoreductase (25). | -1.55 | 4.63E-03 | -1.47 | 8.40E-04 |
| 425107 | *LGALS2* | galectin 2 | *LGALS2* encodes a soluble beta-galactoside binding lectin protein (25). | 2.37 | 2.96E-03 | -3.68 | 3.89E-07 |
| 101750367 | *LOC101750367* | BPI fold-containing family B member 4-like | *LOC101750367* encodes a protein of unknown function. | 2.32 | 2.20E-03 | -4.13 | 9.83E-08 |
| 395683 | *MMP13* | matrix metallopeptidase 13 | *MMP13* encodes a member of the matrix metalloproteinase family. MMPs act in the degradation of extracellular matrix and may be involved in matrix remodeling during tissue growth and morphogenesis. (25,58) | -3.74 | 3.19E-03 | -2.51 | 9.37E-05 |
| 395387 | *MMP9* | matrix metallopeptidase 9 | *MMP9* encodes a matrix metalloproteinase from the zinc-dependent endopepdidases. MMPs act in the degradation of extracellular matrix and may be involved in matrix remodeling during tissue growth and morphogenesis (58). MMP9 may also modulate activity of certain biologically active molecules via cleavage, like angiostatin, gelectin-3, many immune-related molecules (IL-8, IL-1beta, TGFbeta) (58). | -2.55 | 5.23E-08 | -2.41 | 1.31E-06 |
| 423101 | *MUC2* | mucin 2, oligomeric mucus/gel-forming | *MUC2* encodes a mucin glycoprotein associated with the gut lumen barrier but is also found in other tissues, like the gonads (25). While the function of *MUC2* in the gonads is unknown, mucins generally function in the production of protective a protective gel barrier for epithelial cells (59). | -1.71 | 7.47E-03 | -2.93 | 1.01E-04 |
| 374241 | *PI15* | peptidase inhibitor 15 | *PI15* encodes a protein associated with trypsin inhibition (25). | 3.22 | 3.11E-06 | -2.14 | 2.27E-03 |
| 418356 | *PLA2G10L* | phospholipase A2 group X-like | *PLA2G10L* encodes a protein of unknown function. | 3.77 | 6.29E-03 | -3.37 | 2.11E-04 |
| 420203 | *RALYL* | RALY RNA binding protein like | Information is limited on *RALYL*, which encodes a protein that may be associated with RNA and protein binding based on its affiliated GO terms. | -2.36 | 1.69E-03 | 1.28 | 3.28E-04 |
| 427845 | *SLC26A4* | solute carrier family 26 member 4 | *SLC26A4* encodes the protein, pendrin, which acts in the cell membrane transport of chloride, iodide and bicarbonate (25). It is associated with iodide transport in the thyroid gland and the synthesis of thyroid hormones (60). | 2.11 | 9.66E-05 | -2.02 | 9.11E-04 |
| 418719 | *SLC9A2* | solute carrier family 9 member A2 | *SLC9A2* functions as a sodium-hydrogen exchanger (NHE) which regulate cell pH (25). | 2.76 | 1.31E-04 | -2.41 | 2.48E-05 |
| 423225 | *SPTBN5* | spectrin beta, non-erythrocytic 5 | *SPTBN5* encodes the beta-spectrin non-erythrocytic 5 (beta V) protein (61). While spectrins are typically function in cell structure, beta spectrins downregulation has been linked to the impairment of TGFbeta signaling and cell cycle dysregulation (61). | -2.38 | 6.39E-04 | -2.12 | 1.83E-04 |
| 417006 | *SSPO* | SCO-spondin | *SSPO* encodes a thrombospondin type 1 repeat protein that functions in peptidase inhibitor activity (GO:0030414; (25). | 1.70 | 9.54E-03 | -2.71 | 2.30E-04 |
| 768091 | *TSC22D3* | TSC22 domain family member 3 | *TSC22D3* encodes an anti-inflammatory protein glucocorticoid-induced leucine zipper that is stimulated by glucocorticoids and interleukin-10. It functions in inhibiting inflammation and the immune system. (25) | -1.04 | 6.42E-04 | -1.17 | 3.95E-04 |
| 421702 | *VNN1* | vanin 1 | This gene encodes the Vanin-1 enzyme which is associated with pantetheinase activity (62). Pantothenic acid is critical for CoA synthesis (62). Via this pathway, the antioxidant, cysteamine, is also produced (62). *VNN1* increased expression in response to a stressor in rodents (62). This gene is associated with energy metabolism in the liver and regulates glutathione-related responses to oxidative damage in the thymus (62). *VNN1* may upregulate following stress as oxidative stress regulates its expression via an antioxidant response element (ARE)-like element on its promoter region (62). In a *VNN1* knockout study, mice were irradiated to determine the role of *VNN1* on inflammation and oxidative stress. Following irradiation, *VNN1* knockout mice showed lower inflammatory responses and oxidative damage than wild type mice (62). While little research has been conducted on *VNN1* in the ovaries, it is likely upregulated in response to glucocorticoids and restraint stress due to its role in oxidative stress and the inflammatory response. | -1.59 | 3.18E-04 | -1.66 | 3.08E-03 |
|  |  |  |  | -2.42 | 2.09E-04 |  |  |

**Literature Cited:**

1. Muma NA, Beck SG. Corticosteroids alter G protein inwardly rectifying potassium channels protein levels in hippocampal subfields. *Brain Research* (1999) **839**:331–335. doi:10.1016/S0006-8993(99)01754-0

2. Gregerson KA, Flagg TP, O’Neill TJ, Anderson M, Lauring O, Horel JS, Welling PA. Identification of G protein-coupled, inward rectifier potassium channel gene products from the rat anterior pituitary gland. *Endocrinology* (2001) **142**:2820–2832. doi:10.1210/endo.142.7.8236

3. Christensen HR, Zeng Q, Murawsky MK, Gregerson KA. Estrogen regulation of the dopamine-activated GIRK channel in pituitary lactotrophs: implications for regulation of prolactin release during the estrous cycle. *American Journal of Physiology-Regulatory, Integrative and Comparative Physiology* (2011) **301**:R746–R756. doi:10.1152/ajpregu.00138.2011

4. Austin SH, Word K. “Prolactin,” in *Encyclopedia of Animal Cognition and Behavior*, eds. J. Vonk, T. Shackelford (Cham: Springer International Publishing), 1–4. doi:10.1007/978-3-319-47829-6_446-2

5. Bollag WB. Regulation of aldosterone synthesis and secretion. *Comprehensive Physiology* (2014) **4**:39.

6. Matsumoto A, Masuhara M, Mitsui K, Yokouchi M, Ohtsubo M, Misawa H, Miyajima A, Yoshimura A. CIS, a cytokine inducible SH2 protein, is a target of the JAK-STAT5 pathway and modulates STAT5 activation. *Blood* (1997) **89**:3148–3154.

7. Dif F, Saunier E, Demeneix B, Kelly PA, Edery M. Cytokine-inducible SH2-containing protein suppresses PRL signaling by binding the PRL receptor. *Endocrinology* (2001) **142**:5286–5293. doi:10.1210/endo.142.12.8549

8. Radhakrishnan A, Raju R, Tuladhar N, Subbannayya T, Thomas JK, Goel R, Telikicherla D, Palapetta SM, Rahiman BA, Venkatesh DD, et al. A pathway map of prolactin signaling. *J Cell Commun Signal* (2012) **6**:169–173. doi:10.1007/s12079-012-0168-0

9. Yasukawa H, Sasaki A, Yoshimura A. Negative regulation of cytokine signaling pathways. *Annu Rev Immunol* (2000) **18**:143–164. doi:10.1146/annurev.immunol.18.1.143

10. Furuyashiki T, Narumiya S. Stress responses: the contribution of prostaglandin E2 and its receptors. *Nat Rev Endocrinol* (2011) **7**:163–175. doi:10.1038/nrendo.2010.194

11. Rivest S. How circulating cytokines trigger the neural circuits that control the hypothalamic–pituitary–adrenal axis. *Psychoneuroendocrinology* (2001) **26**:761–788. doi:10.1016/S0306-4530(01)00064-6

12. Oka T. Prostaglandin E2 as a mediator of fever: the role of prostaglandin E (EP) receptors. *Front Biosci* (2004) **9**:3046–3057. doi:10.2741/1458

13. Ko C-Y, Chang W-C, Wang J-M. Biological roles of CCAAT/Enhancer-binding protein delta during inflammation. *J Biomed Sci* (2015) **22**:6. doi:10.1186/s12929-014-0110-2

14. Tong Y, Zhou J, Mizutani J, Fukuoka H, Ren S-G, Gutierrez-Hartmann A, Koeffler HP, Melmed S. CEBPD suppresses prolactin expression and prolactinoma cell proliferation. *Molecular Endocrinology* (2011) **25**:1880–1891. doi:10.1210/me.2011-1075

15. McConnell MJ, Licht JD. “The PLZF gene of t(11;17)-associated APL,” in *Acute Promyelocytic Leukemia*, eds. P. P. Pandolfi, P. K. Vogt (Berlin, Heidelberg: Springer Berlin Heidelberg), 31–48. doi:10.1007/978-3-540-34594-7_3

16. Costoya JA. Functional analysis of the role of POK transcriptional repressors. *Briefings in Functional Genomics and Proteomics* (2007) **6**:8–18. doi:10.1093/bfgp/elm002

17. Wasim M, Carlet M, Mansha M, Greil R, Ploner C, Trockenbacher A, Rainer J, Kofler R. PLZF/ZBTB16, a glucocorticoid response gene in acute lymphoblastic leukemia, interferes with glucocorticoid-induced apoptosis. *The Journal of Steroid Biochemistry and Molecular Biology* (2010) **120**:218–227. doi:10.1016/j.jsbmb.2010.04.019

18. Peppi M, Kujawa SG, Sewell WF. A corticosteroid-responsive transcription factor, promyelocytic leukemia zinc finger protein, mediates protection of the cochlea from acoustic trauma. *Journal of Neuroscience* (2011) **31**:735–741. doi:10.1523/JNEUROSCI.3955-10.2011

19. Losa M, Barzaghi RLA, Mortini P, Franzin A, Mangili F, Terreni MR, Giovanelli M. Determination of the proliferation and apoptotic index in adrenocorticotropin-secreting pituitary tumors. *The American Journal of Pathology* (2000) **156**:245–251. doi:10.1016/S0002-9440(10)64725-6

20. McNicol AM, Carbajo-Perez E. Aspects of anterior pituitary growth, with special reference to corticotrophs. *Pituitary* (1999) **1**:257–268. doi:10.1023/A:1009950308561

21. Knoedler JR, Denver RJ. Krüppel-like factors are effectors of nuclear receptor signaling. *General and Comparative Endocrinology* (2014) **203**:49–59. doi:10.1016/j.ygcen.2014.03.003

22. Bonett RM, Hu F, Bagamasbad P, Denver RJ. Stressor and glucocorticoid-dependent induction of the immediate early gene Krüppel-Like Factor 9: Implications for neural development and plasticity. *Endocrinology* (2009) **150**:1757–1765. doi:10.1210/en.2008-1441

23. Besnard A, Langberg T, Levinson S, Chu D, Vicidomini C, Scobie KN, Dwork AJ, Arango V, Rosoklija GB, Mann JJ, et al. Targeting Kruppel-like Factor 9 in excitatory neurons protects against chronic stress-induced impairments in dendritic spines and fear responses. *Cell Reports* (2018) **23**:3183–3196. doi:10.1016/j.celrep.2018.05.040

24. Filipović D, Zlatković J, Gass P, Inta D. The differential effects of acute vs. chronic stress and their combination on hippocampal parvalbumin and inducible heat shock protein 70 expression. *Neuroscience* (2013) **236**:47–54. doi:10.1016/j.neuroscience.2013.01.033

25. O’Leary NA, Wright MW, Brister JR, Ciufo S, Haddad D, McVeigh R, Rajput B, Robbertse B, Smith-White B, Ako-Adjei D, et al. Reference sequence (RefSeq) database at NCBI: current status, taxonomic expansion, and functional annotation. *Nucleic Acids Res* (2016) **44**:D733–D745. doi:10.1093/nar/gkv1189

26. Sirniö P, Väyrynen JP, Klintrup K, Mäkelä J, Mäkinen MJ, Karttunen TJ, Tuomisto A. Decreased serum apolipoprotein A1 levels are associated with poor survival and systemic inflammatory response in colorectal cancer. *Sci Rep* (2017) **7**:5374. doi:10.1038/s41598-017-05415-9

27. Ganfornina MD, Do Carmo S, Lora JM, Torres-Schumann S, Vogel M, Allhorn M, Gonzlez C, Bastiani MJ, Rassart E, Sanchez D. Apolipoprotein D is involved in the mechanisms regulating protection from oxidative stress. *Aging Cell* (2008) **7**:506–515. doi:10.1111/j.1474-9726.2008.00395.x

28. Roszkowski M, Manuella F, von Ziegler L, Durán-Pacheco G, Moreau J-L, Mansuy IM, Bohacek J. Rapid stress-induced transcriptomic changes in the brain depend on beta-adrenergic signaling. *Neuropharmacology* (2016) **107**:329–338. doi:10.1016/j.neuropharm.2016.03.046

29. Regard JB. Verge: A novel vascular early response gene. *Journal of Neuroscience* (2004) **24**:4092–4103. doi:10.1523/JNEUROSCI.4252-03.2004

30. Jadhav K, Zhang Y. Activating transcription factor 3 in immune response and metabolic regulation. *Liver Research* (2017) **1**:96–102. doi:10.1016/j.livres.2017.08.001

31. Bujalka H, Koenning M, Jackson S, Perreau VM, Pope B, Hay CM, Mitew S, Hill AF, Lu QR, Wegner M, et al. MYRF Is a membrane-associated transcription factor that autoproteolytically cleaves to directly activate myelin genes. *PLoS Biol* (2013) **11**:e1001625. doi:10.1371/journal.pbio.1001625

32. Zhang X, Zhang Q, Zhang J, Qiu L, Yan S, Feng J, Sun Y, Huang X, Lu KH, Li Z. FATS is a transcriptional target of p53 and associated with antitumor activity. *Mol Cancer* (2010) **9**:244. doi:10.1186/1476-4598-9-244

33. Yu M, Du G, Xu Q, Huang Z, Huang X, Qin Y, Han L, Fan Y, Zhang Y, Han X, et al. Integrated analysis of DNA methylome and transcriptome identified CREB5 as a novel risk gene contributing to recurrent pregnancy loss. *EBioMedicine* (2018) **35**:334–344. doi:10.1016/j.ebiom.2018.07.042

34. Lee HK, Chaboub LS, Zhu W, Zollinger D, Rasband MN, Fancy SPJ, Deneen B. Daam2-PIP5K is a regulatory pathway for wnt signaling and therapeutic target for remyelination in the CNS. *Neuron* (2015) **85**:1227–1243. doi:10.1016/j.neuron.2015.02.024

35. Canal M, Romani-Aumedes J, Martin-Flores N, Pérez-Fernández V, Malagelada C. RTP801/REDD1: a stress coping regulator that turns into a troublemaker in neurodegenerative disorders. *Front Cell Neurosci* (2014) **8**:1–8. doi:10.3389/fncel.2014.00313

36. Polman JAE, Hunter RG, Speksnijder N, van den Oever JME, Korobko OB, McEwen BS, de Kloet ER, Datson NA. Glucocorticoids modulate the mTOR pathway in the hippocampus: differential effects depending on stress history. *Endocrinology* (2012) **153**:4317–4327. doi:10.1210/en.2012-1255

37. Brockschnieder D. Ermin, A myelinating oligodendrocyte-specific protein that regulates cell morphology. *Journal of Neuroscience* (2006) **26**:757–762. doi:10.1523/JNEUROSCI.4317-05.2006

38. Meyer RC, Giddens MM, Schaefer SA, Hall RA. GPR37 and GPR37L1 are receptors for the neuroprotective and glioprotective factors prosaptide and prosaposin. *Proc Natl Acad Sci USA* (2013) **110**:9529–9534. doi:10.1073/pnas.1219004110

39. Muroi T, Matsushima Y, Kanamori R, Inoue H, Fujii W, Yogo K. GPR62 constitutively activates cAMP signaling but is dispensable for male fertility in mice. *Reproduction* (2017) **154**:755–764. doi:10.1530/REP-17-0333

40. Wang Q, Zhou Q, Zhang S, Shao W, Yin Y, Li Y, Hou J, Zhang X, Guo Y, Wang X, et al. Elevated Hapln2 expression contributes to protein aggregation and neurodegeneration in an animal model of Parkinson’s disease. *Front Aging Neurosci* (2016) **8**: doi:10.3389/fnagi.2016.00197

41. McManus M, Kleinerman E, Yang Y, Livingston JA, Mortus J, Rivera R, Zweidler-McKay P, Schadler K. Hes4: A potential prognostic biomarker for newly diagnosed patients with high-grade osteosarcoma. *Pediatr Blood Cancer* (2017) **64**:e26318. doi:10.1002/pbc.26318

42. Iwamori T, Iwamori N, Matsumoto M, Ono E, Matzuk MM. Identification of KIAA1210 as a novel X-chromosome-linked protein that localizes to the acrosome and associates with the ectoplasmic specialization in testes. *Biology of Reproduction* (2017) **96**:469–477. doi:10.1095/biolreprod.116.145458

43. Wang J, Fa J, Wang P, Jia X, Peng H, Chen J, Wang Y, Wang C, Chen Q, Tu X, et al. NINJ2– A novel regulator of endothelial inflammation and activation. *Cellular Signalling* (2017) **35**:231–241. doi:10.1016/j.cellsig.2017.04.011

44. Kordaß T, Osen W, Eichmüller SB. Controlling the immune suppressor: Transcription actors and microRNAs regulating CD73/NT5E. *Front Immunol* (2018) **9**:813. doi:10.3389/fimmu.2018.00813

45. Bailey MJ, Cassone VM. Melanopsin expression in the chick retina and pineal gland. *Molecular Brain Research* (2005) **134**:345–348. doi:10.1016/j.molbrainres.2004.11.003

46. Yaffe Y, Hugger I, Yassaf IN, Shepshelovitch J, Sklan EH, Elkabetz Y, Yeheskel A, Pasmanik-Chor M, Benzing C, Macmillan A, et al. The myelin proteolipid plasmolipin forms oligomers and induces liquid-ordered membranes in the Golgi complex. *Journal of Cell Science* (2015) **128**:2293–2302. doi:10.1242/jcs.166249

47. Volodko N, Gordon M, Salla M, Ghazaleh HA, Baksh S. RASSF tumor suppressor gene family: Biological functions and regulation. *FEBS Letters* (2014) **588**:2671–2684. doi:10.1016/j.febslet.2014.02.041

48. Dempsey BR, Rintala-Dempsey AC, Shaw GS. “S100 Proteins,” in *Encyclopedia of Signaling Molecules*, ed. S. Choi (New York, NY: Springer New York), 1–10. doi:10.1007/978-1-4614-6438-9_426-1

49. Kärkkäinen S, van der Linden M, Renkema GH. POSH2 is a RING finger E3 ligase with Rac1 binding activity through a partial CRIB domain. *FEBS Letters* (2010) **584**:3867–3872. doi:10.1016/j.febslet.2010.07.060

50. Boeckers TM, Bockmann J, Kreutz MR, Gundelfinger ED. ProSAP/Shank proteins - a family of higher order organizing molecules of the postsynaptic density with an emerging role in human neurological disease: Role of ProSAP/Shank in PSD organization. *Journal of Neurochemistry* (2002) **81**:903–910. doi:10.1046/j.1471-4159.2002.00931.x

51. Petrova R, Joyner AL. Roles for Hedgehog signaling in adult organ homeostasis and repair. *Development* (2014) **141**:3445–3457. doi:10.1242/dev.083691

52. Li H, Lu Y, Smith HK, Richardson WD. Olig1 and Sox10 interact synergistically to drive myelin basic protein transcription in oligodendrocytes. *Journal of Neuroscience* (2007) **27**:14375–14382. doi:10.1523/JNEUROSCI.4456-07.2007

53. Mariotti S, Beck-Peccoz P. “Physiology of the Hypothalamic-Pituitary-Thyroid axis,” in *Endotext*, eds. K. R. Feingold, B. Anawalt, A. Boyce, G. Chrousos, K. Dungan, A. Grossman, J. M. Hershman, G. Kaltsas, C. Koch, P. Kopp, et al. (South Dartmouth (MA): MDText.com, Inc.). Available at: http://www.ncbi.nlm.nih.gov/books/NBK278958/ [Accessed January 23, 2020]

54. Bosio A, Binczek E, Le Beau MM, Fernald AA, Stoffel W. The human gene CGT encoding the UDP-galactose ceramide galactosyl transferase (Cerebroside Synthase): Cloning, characterization, and assignment to human Chromosome 4, Band q26. *Genomics* (1996) **34**:69–75. doi:10.1006/geno.1996.0242

55. Ma KL, Ruan XZ, Powis SH, Chen Y, Moorhead JF, Varghese Z. Inflammatory stress exacerbates lipid accumulation in hepatic cells and fatty livers of apolipoprotein E knockout mice. *Hepatology* (2008) **48**:770–781. doi:10.1002/hep.22423

56. Heinke J, Kerber M, Rahner S, Mnich L, Lassmann S, Helbing T, Werner M, Patterson C, Bode C, Moser M. Bone morphogenetic protein modulator BMPER is highly expressed in malignant tumors and controls invasive cell behavior. *Oncogene* (2012) **31**:2919–2930. doi:10.1038/onc.2011.473

57. Datler C, Pazarentzos E, Mahul-Mellier A-L, Chaisaklert W, Hwang M-S, Osborne F, Grimm S. CKMT1 regulates the mitochondrial permeability transition pore in a process that provides evidence for alternative forms of the complex. *Journal of Cell Science* (2014) **127**:1816–1828. doi:10.1242/jcs.140467

58. Swain N, Pathak J, Patel S, Hosalkar RM. “MMP-9,” in *Encyclopedia of Signaling Molecules*, ed. S. Choi (New York, NY: Springer New York), 1–6. doi:10.1007/978-1-4614-6438-9_102000-1

59. Duraisamy S, Kufe T, Ramasamy S, Kufe D. Evolution of the human MUC1 oncoprotein. *Int J Oncol* (2007) doi:10.3892/ijo.31.3.671

60. Bizhanova A, Kopp P. Controversies concerning the role of pendrin as an apical iodide transporter in thyroid follicular cells. *Cell Physiol Biochem* (2011) **28**:485–490. doi:10.1159/000335103

61. Czogalla A. “Spectrin,” in *Encyclopedia of Signaling Molecules*, ed. S. Choi (New York, NY: Springer New York), 1–7. doi:10.1007/978-1-4614-6438-9_101871-1

62. Berruyer C, Martin FM, Castellano R, Macone A, Malergue F, Garrido-Urbani S, Millet V, Imbert J, Dupre S, Pitari G, et al. Vanin-1-/- mice exhibit a glutathione-mediated tissue resistance to oxidative stress. *Molecular and Cellular Biology* (2004) **24**:7214–7224. doi:10.1128/MCB.24.16.7214-7224.2004
